# Combining ability studies of grain Fe and Zn contents of pearl millet (*Pennisetum glaucum* L.) in West Africa

**DOI:** 10.3389/fpls.2022.1027279

**Published:** 2023-01-06

**Authors:** Bassirou Sani Boubacar Gaoh, Prakash I. Gangashetty, Riyazaddin Mohammed, Issoufou Kassari Ango, Daniel Kwadjo Dzidzienyo, Pangirayi Tongoona, Mahalingam Govindaraj

**Affiliations:** ^1^ Pearl Millet Breeding, International Crops Research Institute for the Semi-Arid Tropics, Niamey, Niger; ^2^ West African Centre for Crop Improvement, College of Basic and Applied Sciences, University of Ghana, Legon, Ghana; ^3^ Pigeon Pea Breeding, International Crops Research Institute for the Semi-Arid Tropics, Patancheru, India; ^4^ Department of Rainfed Crop Production (DCP), Institute National de la Recherche Agronomique du Niger, Maradi, Niger; ^5^ HarvestPlus, Alliance of Bioversity International and the International Center for Tropical Agriculture (CIAT), Cali, Colombia

**Keywords:** *Pennisetum glaucum* L., micronutrient malnutrition, combining ability, biofortification, grain Fe and Zn, diallel analysis

## Abstract

Micronutrient malnutrition is a major challenge in Africa, where half a million children die each year because of lack of micronutrients in their food. Pearl millet is an important food and fodder crop for the people living in the Semi-Arid regions of West Africa. The present study was conducted to determine the stability, combining ability, and gene action conditions of the high level of Fe and Zn content in grain and selected agronomic traits. Hence, eight genotypes were selected based on the availability of grain Fe and Zn contents and crossed in a full diallel mating design. Progenies from an 8 × 8 diallel mating along with the parents were evaluated in an alpha lattice design with three replications in three locations for two years. The parental lines Jirani, LCIC 9702 and MORO, had positive significant general combining ability (GCA) effects for grain Fe concentration, while Jirani and MORO had positive significant GCA effects for grain Zn concentration. For the specific combining ability (SCA), among the 56 hybrids evaluated, only the hybrids LCIC 9702 × Jirani and MORO × ZANGO had positive significant SCA effects for grain Fe concentration across locations, and for grain Zn concentration, the hybrids Gamoji × MORO, LCIC 9702 × Jirani, and ICMV 167006 × Jirani had positive significant SCA effects. The reciprocal effects were significant for grain Zn concentration, grain yield, flowering time, plant height, test weight, and downy mildew incidence, suggesting that the choice of a female or male parent is critical in hybrid production. Grain Fe and Zn concentration, flowering time, plant height, panicle length, panicle girth, panicle compactness, and downy mildew incidence were found to be predominantly under additive gene action, while grain yield and test weight were predominantly under non-additive gene action. A highly positive correlation was found between grain Fe and Zn concentrations, which implies that improving grain Fe trait automatically improves the grain Zn content. The stability analysis revealed that the hybrid ICMV 167006 × Jirani was the most stable and high-yielding with a high level of grain Fe and Zn micronutrients.

## Introduction

Micronutrient malnutrition affects around 2 billion people worldwide, and most countries in West Africa have serious levels of hunger (Global Hunger Index, 2019). The people living in West and Central Africa (WCA) do not have access to nutritionally balanced food products. As a result, they suffer from micronutrient malnutrition or “hidden hunger” (Badu‐Apraku and Fakorede, 2017). Pearl millet (*Pennisetum glaucum* L.) is the sixth most important cereal globally, and it is the staple food for the people living in drought-prone areas of Africa. It is a drought-tolerant cereal predominantly grown in the semi-arid regions of West Africa, where other cereals fail to produce. The diet in West Africa is mostly based on pearl millet. This cereal occupies a large proportion of the cereals grown and consumed because of its hardiness and ability to withstand high arid temperatures. In addition, farmers grow pearl millet because it is the most reliable cereal crop cultivated under short growing seasons and low rainfall conditions (Taylor, 2004; Uzoma et al., 2010; Srivastava et al., 2022). The drought and heat-tolerant features of pearl millet make this crop a priority crop for fighting hunger in WCA.

In West Africa, the presence of highly variable environments, nutrient-poor soils, and abiotic and biotic constraints affect the performance of cultivars (Sattler et al., 2019; Sharma et al., 2021). There are several biotic and abiotic constraints affecting the grain yields in pearl millet. Downy mildew (*Sclerospora graminicola*), millet head miner (*Heliocheilous albipunctella*), striga (*Striga hermonthica*), drought, and high temperatures are the major biotic and abiotic constraints affecting the grain yields of pearl millet. In addition, the pearl millet varieties grown in Sub-Saharan Africa are mostly OPVs with limited yield potential. In 2020, pearl millet was grown on 13.75 m ha in West Africa, which was 42.83% of the total pearl millet area grown in the world and has recorded lower grain yields of 740 kg/ha, which is far less than the world average yield based on FAO statistics (FAOSTAT, 2020). In Niger during the 2021 rainy season, pearl millet yields were estimated at 349 kg/ha (National Institute of Statistics in Niger, 2022). However, in India, due to the rapid introduction of hybrids in all regions that replaced the cultivation of landraces and OPVs, pearl millet production shifted from 288 kg/ha in 1950–1951 to 1,255 kg/ha in 2014–2015 (Charyulu et al., 2016). In 2020, productivity of 1,243 kg/ha was reported (Sanjana Reddy et al., 2021). Therefore, it is important to identify the best-yielding genotypes that display high stability for grain yield, Fe, and Zn content across the region.

Many studies have shown the positive impact of the consumption of biofortified crops and the results of these studies were also similar to iron fortification and supplementation strategies to fight against hidden hunger (Kodkany et al., 2013; Saltzman et al., 2013; Haas, 2014; Finkelstein et al., 2015; Foley et al., 2021). Recently, Africa’s first biofortified pearl millet variety, CHAKTI, was released in Niger (Jayashree and Meyer, 2018). This variety is gaining importance in the farming communities in Niger, Mali, Burkina Faso, and Senegal because of its extra early maturity, high yield, and high grain Fe and Zn content (Mavindidze, 2020). Efforts are underway at the ICRISAT and National Agricultural Research systems (NARs) breeding programs in West Africa to acquire genetic information on these traits for pearl millet hybrid breeding programs to efficiently develop improved pearl millet varieties with high grain Fe and Zn content. However, there is only limited information on genetic variability and heterosis for grain Fe and Zn content in West African pearl millet varieties.

The use of stable hybrids rich in grain Fe and Zn concentration and with high grain yield, which correspond to the needs of farmers, is an appropriate and sustainable strategy for resource-poor farmers to alleviate hidden hunger in Niger. Hence, the present study was undertaken to assess the variability and heritability of grain Fe and Zn concentrations, grain yield, flowering time, panicle length, and plant height and selected agronomic traits in pearl millet, determine the combining abilities, estimate the heterosis, and determine the stability of pearl millet hybrids for high grain Fe and Zn concentration and high grain yield.

## Materials and methods

### Field evaluation

Field evaluations were performed at three locations at the ICRISAT Sadoré (13°14’40.66”N 2°16’38.92”E) research station, the Institute National de la Recherche Agricole du Niger (INRAN)-Tara (11°55’24.1”N 3°20’03.5”E) research station, and the INRAN-Maradi (13°27’43.2”N 7°06’32.9”E) research station in Niger during the 2017 and 2018 cropping seasons.

### Genetic materials

The experimental material consists of diverse parents selected from previous information on the grain Fe and Zn contents, agronomic, and morphological traits. It consisted of the two landraces Gamoji and MORO and six open-pollinated varieties (OPVs) ICMV IS 89305, Jirani, LCIC 9702, ICMV 167006, and ZANGO (**Table 1**). The genotypes CHAKTI, GB 8735, ICMV IS 92222, SOSAT-C88, ICMV 167005, and ICMV IS 99001, were used as controls for this trial. To generate, under the field conditions, enough seeds for the multilocation and multiyear trials, each parental line was sown in a four-row plot (female) followed by the other parental lines in a two-row plot (pollen source), such as P1 (four rows)–P2 (two rows); P1 (four rows)–P3 (two rows); P1 (four rows)–P4 (two rows); P8 (four rows)–P6 (two rows); and P8 (four rows)–P7 (two rows). In addition, two nurseries used as pollen sources were planted at two different dates (10-day intervals), composed of all eight parental lines sown each in an eight-row plot (seven hills per row). An average of 19 crosses for each combination was performed, which allowed the generation of 28 direct crosses and 28 reciprocals.

**Table 1:** Parental lines and checks used in the 8x8 full diallel.

S no	Genotypes	Origin/description
1	Gamoji	Released in Niger, developed by Institut National de la Recherche Agronomique du Niger (INRAN), purified from the landrace Gamoji of Zinder region
2	LCIC 9702	Released by Lake Chad Research Institute (LCRI), improved variety from Nigeria with GB 8735 parentage
3	ICMV IS 89305	Released variety in 1989 bred by ICRISAT-Niger from a cross of 3/4 HK B-78, Souna-3 and CIVT
4	ICMV 167006	Released in Niger, developed by ICRISAT-Niger in 2005, selected after many generations of purification of the variety B9_Tabi of Boni in Mali.
5	MORO	Released in Niger by INRAN in 1985, purified from the landrace Moro of Diffa region
6	Jirani	Released by the Lake Chad Research Institute (LCRI), Bred by ICRISAT- Niger in 2013, bred from iniadi landrace of Burkina Faso
7	HKP	HKP Released in Niger. Improved population developed by INRAN in 1978.
8	ZANGO	Released in Niger. Developed by INRAN in 1986, purified from the landrace Zongo
Checks	CHAKTI	Released in West Africa, Developed by ICRISAT-Niger in 2018, bred by intra population selection in ICTP 8203. It's the first Fe biofortified variety released in West Africa
	GB 8735	Released in West Africa, developed by ICRISAT-Niger in 1987, bred by recurrent selection from a cross between Iniadi and Souna landraces
	ICMV IS 92222	Released in West Africa, developed by ICRISAT-Niger in 1992, obtained from the landrace Haïné Kiré after a purification and selection of S1
	SOSAT-C88	Released in West Africa, developed by ICRISAT-Niger in 1988, bred by recurrent selection from a cross in Mali between Souna and Sanio Mali landraces
	ICMV 167005	Released in West Africa, developed by ICRISAT-Niger in 2005, purified from the landrace through intra population selection methods.
	ICMV IS 99001	Released in West Africa, developed by ICRISAT-Niger in 1987, bred by recombination of S1 from gridded mass selection of HKP

The 56 crosses, along with the parents and selected checks, were sown in an alpha lattice design in three replications, across three locations. The test material was sown in two rows of 4.8 m in length each, with a distance of 0.75 m between the rows. In addition, there was spacing between the hills of 0.8 m *i.e.*, seven hills per row. The test plots were thinned to two plants per hill 12 days after emergence (DAE). A basal application of mineral fertilizer at 100 kg/ha was carried out during the field preparation. Weeding of the test plots was carried out when necessary. The test plots were micro-dosed with urea (3 g per hill) at 30 DAE. The trial was grown under rain-fed conditions and no external irrigation was provided. Normal agronomic practices were followed during the growth of the trial.

### Data collection

Data was collected for downy mildew incidence (DM), flowering time in days (FLO), plant height in cm (Pht), panicle length in cm (PL), panicle girth (PG), panicle compactness (Pcom), thousand seed weight in g (1000 sdw), grain yield in t/ha (GY) following the descriptor IBPGR/ICRISAT et al., 1993, the grain Fe content (GFe) and grain Zn content (GZn) in ppm were measured using the ED-XRF (energy-dispersive X-ray fluorescence) spectrometer at the ICRISAT-Sadoré research station. The ED-XRF conditions used for the analysis were as suggested by Paltridge et al. (2012).

### Data analysis

Diallel analysis was carried out based on method I and model 1 of Griffing (1956), and the GGE biplot analysis was performed using Plant Breeding Tools (PBTools) version 1.3 (IRRI, 2013–2020). To determine the significance of the general combining ability (GCA), specific combining ability (SCA), and reciprocal combining ability (RCA), the t-test was used according to Griffing (1956). To determine the relative importance of GCA and SCA, the predictability ratio was calculated according to Baker (1978). The performance *per se* of parental lines and hybrids and the interrelation among grain Fe and Zn contents and agronomic traits were analyzed using Fonseca software (VSN International, 2015). Mid-parent heterosis and better-parent heterosis were calculated following Fonseca and Patterson (1968). The significance of F_1_ hybrids from mid-parent and better-parent heterosis was determined using a t-test (Wynne et al., 1970; Iqbal et al., 2009). Narrow and broad sense heritabilities were calculated according to Teklewold and Becker (2005).

## Results

### Performance *per se* of parents and hybrids

The mean performance of grain Fe concentration over the locations and years among the parents ranged from 36.0 ppm (HKP) to 56.6 ppm (Jirani), while among the hybrids (direct crosses and reciprocal crosses) the mean ranged from 35 ppm (HKP × ZANGO) to 57.5 ppm (Jirani × LCIC 9702) (**Table 2**), whereas the mean of grain Zn concentration among the parents and hybrids was 37.4 ppm. The average grain Zn concentration among the parental lines varied from 33.5 ppm (HKP) to 49.0 ppm (Jirani) and among the hybrids from 30.9 ppm (HKP × ICMV 167006) to 49.0 ppm (Jirani × LCIC 9702). For grain yield, the hybrids (direct and reciprocal crosses) were more productive compared to the parents across the six environments. The grain yield among the parents ranged from 0.6 t/ha for LCIC 9702 to 1.1 t/ha for ICMV IS 89305, while among the hybrids the average grain yield varied from 1.2 t/ha for MORO × Jirani to 2.6 t/ha for ICMV IS 89305 × ICMV 167006. Among the top seven hybrids, the hybrid ICMV 167006 × HKP was the lowest yielding (with 2.3 t/ha) and the hybrid ICMV IS 89305 × ICMV 167006 was the highest yielding genotype (with 2 t/ha). All the top seven hybrids have the genotype ICMV 167006 as a parent. Among the 64 genotypes (eight parents and 58 hybrids), the earliest genotype was the hybrid Jirani × LCIC 9702 which flowered after 48.7 days, while the parental line ICMV 167006 was the latest genotype to flower after 62.2 days. For plant height, the mean performance across environments was 209.9 cm. In the pooled analysis, the mean plant height among the parents varied from 162.1 cm for Jirani to 216.1 cm for ICMV 167006, and among the hybrids, it varied from 173.4 cm for LCIC 9702 × Jirani to 238.5 cm for ICMV 167006 × HKP. Among the five tallest hybrids, four hybrids have the genotype ICMV 167006 in their parentage, while the five shortest hybrids have Jirani as a parent. The panicle length across sites ranged from 22.6 cm for Jirani to 51.3 cm for HKP among the parents, while for the hybrids it ranged from 24.8 cm for Jirani × LCIC 9702 to 55.8 cm for ZANGO × ICMV IS 89305. The variation of panicle girth across environments was similar among parents and hybrids. Among the parents, it ranged from 6.7 cm for MORO to 10.2 cm for Gamoji, while among the hybrids it ranged from 6.7 cm for ICMV 167006 × MORO to 10 cm for Gamoji × LCIC 9702. Gamoji and Jirani both had a panicle girth across environments that were above the overall mean of 8.1 cm. The compactness of the panicle was scored from loose (3) to very compact (1). Among the parental lines, the genotypes ICMV 167006 and MORO had the best compact panicle, and one or both genotypes were also in the parentage of the top ten hybrids with very compact panicles. Across sites, the parental line Gamoji had the loosest panicle. For test weight, the mean performance of the parents over the environments ranged from 9.2 g (ICMV 167006) to 11.1 g (LCIC 9702) in the parental lines, while among hybrids it ranged from 9.9 g (ICMV 167006 × MORO) to 12.3 g (HKP × Jirani). The downy mildew incidence was the lowest for the hybrid ICMV IS 89305 × ZANGO (2%) while it was the highest for the hybrid HKP × LCIC 9702 (27.7%).

**Table 2:** Pooled performance *per se* of grain iron and zinc content and agronomic traits of pearl millet genotypes (8 parents and 56 hybrids).

Entry	Pedigree	GFe	GZn	FLO	Pht	PL	PG	GY	Pcom	1000sdw	DM
1	Gamoji x HKP	39.2	33.2	59.8	211.9	44.1	8.8	1.6	2.4	11.3	11.7
2	Gamoji x ICMV IS 89305	38.8	34.3	60.6	207.6	41.1	9.1	1.9	2.2	10.8	6.6
3	Gamoji x ICMV 167006	41.7	36.7	57.9	227.7	37.3	8.7	2.5	1.6	11.3	10.3
4	Gamoji x Jirani	45.1	39.1	53.1	187.2	33.6	9.6	1.8	2.5	12	12.9
5	Gamoji x LCIC9702	43.1	36.5	57	195.1	36.2	10	1.7	2.3	11.7	5.9
6	Gamoji x MORO	44.4	41.2	57.7	214.6	34.1	8.6	2.3	1.7	11.1	10.2
7	Gamoji x ZANGO	39.2	33.4	59	229.1	51.7	9.2	1.6	2.4	12.2	9.6
8	HKP x Gamoji	35.7	32.9	60.4	230.1	48.9	9	2	2.3	11.2	12.9
9	HKP x ICMV IS 89305	38.1	33.5	59.8	221.5	47.8	7.6	2	1.8	10.7	6
10	HKP x ICMV 167006	36.9	30.9	58.8	234	44	7.4	2.5	1.7	11.3	15.4
11	HKP x Jirani	44.2	38.9	53.4	205.7	39.9	8.4	1.6	2.1	12.3	17.9
12	HKP x LCIC 9702	40.7	34.8	56.6	208.3	41.7	8.4	1.9	1.9	11.4	27.7
13	HKP x MORO	41.6	38	56.4	225.5	37.9	7.4	1.9	1.4	10.9	12.8
14	HKP x ZANGO	35	32.3	59.1	233	53.3	7.9	2	2.3	11.1	12.6
15	ICMV IS 89305 x Gamoji	40	33.9	58.8	223.4	41.9	8.8	1.9	2.1	11.2	10.9
16	ICMV IS 89305 x HKP	36.6	32.4	59.8	231.8	49.1	8.1	1.8	1.7	11.1	3.9
17	ICMV IS 89305 x ICMV 167006	42.8	36.6	59.8	235.6	39.1	8	2.6	1.2	10.5	6.1
18	ICMV IS 89305 x Jirani	49.7	41.7	53.7	199.3	33.5	8	1.5	2	10.2	13.2
19	ICMV IS 89305 x LCIC 9702	42.2	36.3	55.6	199.8	37.4	8.2	1.9	2	11.4	7.6
20	ICMV IS 89305 x MORO	41.5	36.6	57.7	206.9	33	7.8	1.9	1.7	10.3	9.9
21	ICMV IS 89305 x ZANGO	36.5	31.9	59.7	236.6	54.3	8.9	2.1	2.3	11.3	2
22	ICMV 167006 x Gamoji	41.4	35.4	60.3	209.4	34.7	8.5	1.4	1.8	11.4	13.8
23	ICMV 167006 x HKP	38.2	32.5	59.7	238.5	44.6	7.4	2.3	1.3	10.8	12.2
24	ICMV 167006 x ICMV IS 89305	40.5	34.5	59.7	223.8	34.2	9.4	2.2	1.7	10.9	3.3
25	ICMV 167006 x Jirani	48.9	42.3	54.2	200.1	28.5	7.6	1.9	1.5	12.2	16.8
26	ICMV 167006 x LCIC 9702	44.8	35.8	55.1	216.2	32.3	9.3	2.5	1.5	11.3	9.4
27	ICMV 167006 x MORO	45.6	38.8	57.8	205.2	27.8	6.7	2	1.3	10	9.9
28	ICMV 167006 x ZANGO	38.4	32.9	58.1	236.9	41.6	7.4	1.8	1.2	11.8	4.8
29	Jirani x Gamoji	49	42.4	55.9	199.9	31	9.3	1.4	2.7	11	7.7
30	Jirani x HKP	45.3	38	53.3	197.3	36.3	7.8	1.6	2.3	12	16.7
31	Jirani x ICMV IS 89305	48.4	41.7	53.9	192.2	31.8	7.8	1.3	1.7	11.5	8.2
32	Jirani x ICMV 167006	53	45.2	54.5	207.7	29.4	7.6	1.6	1.7	11.2	11.1
33	Jirani x LCIC 9702	57.5	49	48.7	176.8	24.8	8.7	1.2	2.3	11.7	14.8
34	Jirani x MORO	51.4	43.4	49.7	182.9	27.1	7.7	2.1	1.7	10.2	18.7
35	Jirani x ZANGO	42.3	37.5	54.8	211.1	38.1	8.1	1.5	2.3	12.2	12.9
36	LCIC 9702 x Gamoji	43.1	35	56.5	194.6	36.3	9.1	1.9	2.2	11.7	10.4
37	LCIC 9702 x HKP	40.3	34.7	56.4	213.3	44.7	8.2	2.1	2.3	11.6	11.1
38	LCIC 9702 x ICMV IS 89305	43.9	35.8	55.9	202.7	40.4	8.1	1.7	1.9	11.7	7.3
39	LCIC 9702 x ICMV 167006	46.1	37.4	57.6	215.8	32.7	7.8	2.4	1.4	11.9	13.7
40	LCIC 9702 x Jirani	50.9	40.2	50.6	173.4	25.2	9.2	1.3	2.5	11.4	11.8
41	LCIC 9702 x MORO	47.1	40.2	53.2	198.3	27.4	7.6	1.6	1.4	10.7	9.2
42	LCIC 9702 x ZANGO	41.1	35.5	54.5	204.4	43.9	8.4	1.7	2.4	11.5	8.9
43	MORO x Gamoji	45.6	41.4	57.2	218.2	36.6	8.4	2.1	1.8	10.6	2.3
44	MORO x HKP	40.8	36.8	57.7	214.9	39.8	7.6	1.7	1.7	11	9.4
45	MORO x ICMV IS 89305	44.7	39.3	56.6	219.3	36.8	7.4	1.9	1.5	11.2	3.4
46	MORO x ICMV 167006	45.9	38.7	57.5	209.6	30.7	7.3	2.3	1.3	10.7	11
47	MORO x Jirani	53.3	45.3	50.7	183.4	25.2	7.6	1.2	1.9	10.7	18
48	MORO x LCIC 9702	48.6	38.8	51.7	206.1	30.4	7.7	2.1	1.3	11.5	7.9
49	MORO x ZANGO	44.2	39.3	56.8	217.1	42.2	7.6	1.7	1.7	11.2	5.6
50	ZANGO x Gamoji	38.5	33.5	58.9	229.8	44.8	8.7	2.1	1.9	11.1	4.8
51	ZANGO x HKP	38.6	35.5	58.8	233.2	54	8.1	1.7	2.1	12	4.8
52	ZANGO x ICMV IS 89305	37.9	33.1	58.1	215.7	64.4	8.3	1.7	2.1	10.4	5.4
53	ZANGO x ICMV 167006	40.8	37.1	58.7	230.3	42.9	7.6	2	1.6	11.6	6
54	ZANGO x Jirani	42.4	37.9	53.1	206.4	41.1	8.2	1.4	2.5	11.4	10.8
55	ZANGO x LCIC 9702	41	35.4	53.7	205.8	41.9	8.4	1.7	2.1	11.7	6.9
56	ZANGO x MORO	45.3	39.9	56.2	219.9	40.1	7.4	1.3	1.6	10.7	11.7
57	MORO	51.4	45.1	57.3	192.3	26.6	6.7	1.1	1.3	9.8	7.2
58	Gamoji	39.5	34.8	60.4	188.7	36.7	10.2	1	2.8	10.8	12.2
59	ICMV IS 89305	39.4	34.2	60.8	207.7	38.4	8.1	1.1	2.2	9.7	4.7
60	ICMV 167006	44.9	38.9	62.2	216.1	29.6	6.8	0.7	1.3	9.2	12.2
61	HKP	36	33.5	60.2	213.6	51.3	7.4	1	2.4	10.7	13.3
62	ZANGO	38.5	35.6	61.2	215.2	43.7	7.6	0.9	1.8	9.5	4.9
63	LCIC9702	47.5	37	54.7	162.7	27.8	7.8	0.6	2.4	11.1	8.5
64	Jirani	56.6	49	50.4	162.1	22.6	8.4	0.8	2.4	9.9	18.3
	**Grand mean**	43.3	37.4	56.7	209.9	38.1	8.2	1.7	1.9	11.1	10.1
	*e.s.e.*	3.4	3.2	1.3	13.1	4.8	0.9	0.4653	0.3268	0.6449	5.894
	*v.r.*	13.6***	9.4***	32.6***	10.8***	18.4***	4.2***	5.9***	9.8***	6.8***	3.8***
	*cv%*	13.6	15	4.1	10.8	21.9	19.1	46.8	29.5	10.1	100.7
	*l.s.d.*	9.4	8.9	3.7	36.4	13.4	2.5	1.2917	0.9072	1.7904	16.363

e.s.e. Standard errors of means.

v.r. variance ratio.

cv% coefficients of variation.

l.s.d. Least significant differences of means (5% level).

***, F value significant at P <0.001.

GFe, Grain iron density in ppm; GZn, Grain zinc density in ppm; FLO, flowering time; Pht, plant height in cm; PL, panicle length in cm; PG, panicle girth in cm; GY, grain yield (t/ha); Pcom, panicle compactness (score); 1000sdw, thousand seed weight in g and DM, downy mildew incidence in percentage.

### Estimates of variance and genetic variance components

The pooled analysis of variance indicated a highly significant difference (P <0.01) among environments for all studied characters, except for grain Fe concentration, which was significant at P <0.05 (**Table 3**). Similarly, for crosses (parents, direct crosses, and reciprocal crosses), all studied characters showed highly significant (P <0.01) differences. There were significant differences among general combining abilities for all traits except for downy mildew incidence, which was non-significant. For grain Fe concentration, panicle length, panicle girth, and compactness of panicle reciprocal effects were non-significant, while for grain Zn concentration and downy mildew incidence maternal effects were significant (P <0.05), and consequently reciprocal effects were significant for these traits. The maternal and non-maternal effects were both significant for flowering time, plant height, and test weight, whereas for grain yield only the non-maternal effects were significant at P <0.05.

**Table 3:** Mean squares of crosses and combining ability effects of grain iron and zinc density and agronomic traits evaluated in 56 F1 families and 8 parents combined from six environments (three locations and two rainy seasons).

	Df	Grain Fe content	Grain Zn content	Flowering time	Plant height	Panicle length	Panicle girth	Grain yield	Compactness of panicle	Thousand seed weight	Downy mildew incidence
Env	5	667.86*	3925.06**	3694.99**	48719.26**	2670.93**	48.92**	251.36**	14.82**	232.28**	5086.37**
Rep(Env)	12	179.81**	223.88**	60.41**	2069.78**	85.52ns	5.51**	9.33**	0.24ns	1.32ns	233.45**
Crosses	63	468.76**	293.71**	174.16**	6221.81**	1287.45**	10.19**	4.07**	3.18**	9.24**	396.08**
Crosses x Env	315	38.36*	35.88**	9.83**	973.01**	73.42ns	2.63ns	1.01**	0.49**	1.72ns	215.12**
GCA	7	3799.58**	2195.63**	1388.33**	36986.8**	10061.04**	65.99**	7.02**	22.16**	23.23**	1876.31ns
SCA	28	58.19*	61.85**	31.86**	2715.4**	278.73**	3.84*	5.62**	1.14**	10.53**	189.66**
REC	28	46.64ns	50.09*	12.92**	2036.97**	102.76ns	2.59ns	1.78**	0.49ns	4.45**	232.44**
MAT	7	68.56ns	64.95*	11.97*	1636.3**	97.46ns	2.92ns	1.88**	0.32ns	4.18*	374.12*
NONM	21	39.33ns	45.13ns	13.23**	2170.53**	104.53ns	2.48ns	1.74**	0.54ns	4.54**	185.21ns
GCA x E	35	64.2**	74.73**	37.65**	1587.47**	78.34ns	3.79*	1.11**	1.44**	1.8ns	1039.73**
SCA x E	140	35.64ns	30.37ns	6.75**	1078.85**	69.58ns	2.47ns	1.3**	0.33ns	1.82ns	102.13ns
REC x E	140	34.62ns	31.68ns	5.97**	713.56**	76.03ns	2.5ns	0.69*	0.4*	1.59ns	121.96ns
MAT x E	35	33.68ns	28.1ns	4.9ns	526.82ns	63.04ns	3.26ns	0.47ns	0.41ns	1.66ns	139.56ns
NONM x E	105	34.93ns	32.88ns	6.33**	775.81**	80.36ns	2.25ns	0.76**	0.4ns	1.57ns	116.1ns
Residuals	753	32.32	28.76	4.51	516.29	69.77	2.43	0.55	0.32	1.62	102.33

ns, non-significant; *, significant at P<0.05; **, significant at P <0.01.

The cross–environment interactions were significant for all traits apart from panicle length, panicle girth, and test weight. The mean squares of the general combining ability × environment (GCA × E) interaction were highly significant (P <0.001) for all traits except for panicle girth (significant at P <0.05) and for panicle length and test weight (which were non significant). The specific combining ability × environment (SCA × E) interaction was non-significant for all traits except for flowering time, plant height, and grain yield. For the interaction of reciprocal effects and environment (REC × E) only flowering time was significant at P <0.05, and the interaction of non-maternal effects and environment (NONM × E) was significant at P <0.01.

**Figure 1:**
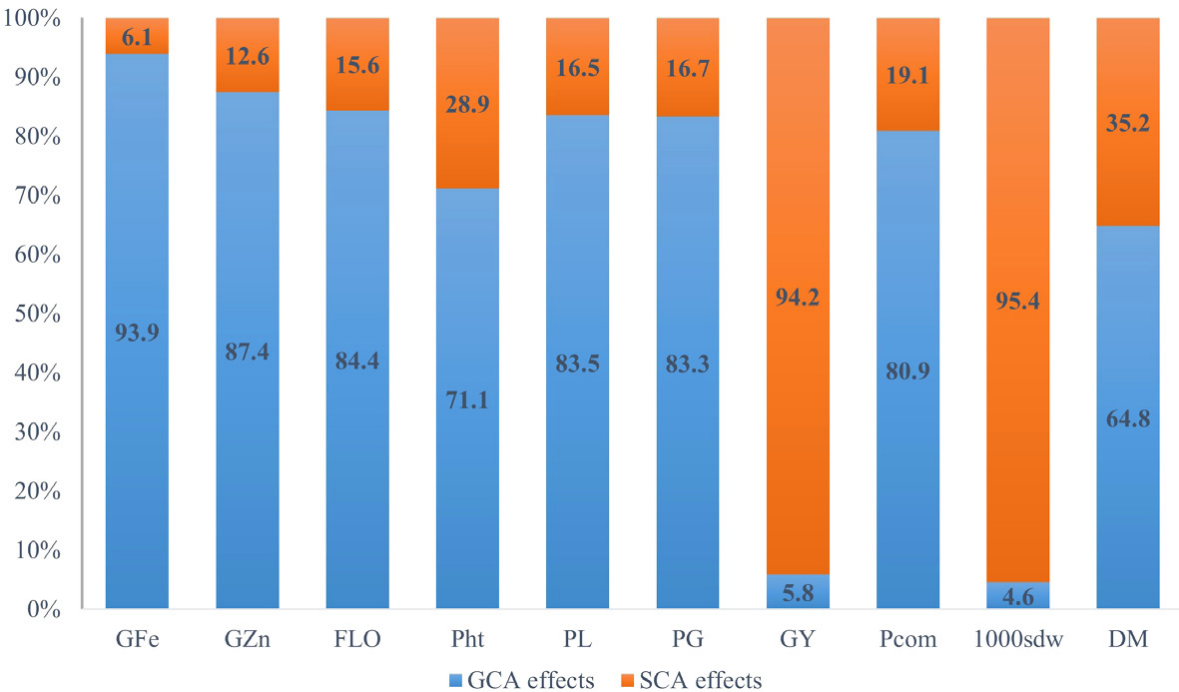
Relative proportions of GCA and SCA for gain iron content (GFe), grain zinc content (GZn), and selected agronomic traits in an 8 x 8 full diallel analysis, averaged over six environments (three locations and two rainy seasons) (GFe, Grain iron density; GZn, Grain zinc density; FLO, flowering time; Pht, plant height; PL, panicle length; PG, panicle girth; GY, grain yield; Pcom, panicle compactness; 1000sdw, thousand seed weight, and DM, downy mildew incidence).

The overall variance due to general combining ability (Ơ^2^_GCA_) was predominantly higher than the variance of specific combining ability (Ơ^2^_SCA_) for all traits studied, except for grain yield and test weight (**Table 4** and **Figure 1**). For grain Fe concentration, the GCA variance was 15 times higher than the SCA variance, while for Zn grain concentration, the GCA variance was around seven times higher than the SCA variance. For grain yield and test weight, the SCA variance was greater than the GCA variance by 16 and 20 times. The predictability ratio, was close to 1 for grain Fe and Zn concentration, flowering time, panicle length, and panicle girth. The predictability ratio was very low for grain yield (0.13) and test weight (0.07). The narrow sense heritability and broad sense heritability were very high for grain Fe and Zn concentrations, flowering time, panicle length, panicle girth, and panicle compactness. However, the narrow-sense heritability was low for grain yield and test weight.

**Table 4:** Estimates of variance and genetic variance components.

Variance components	Grain Fe content	Grain Zn content	Flowering time	Plant height	Panicle length	Panicle girth	Grain yield	Compactness of panicle	Thousand seed weight	Downy mildew incidence
Ơ^2^_GCA_	229.09	128.66	83	1982.41	593.97	3.55	0.21	1.24	0.2	90.13
Ơ^2^_SCA_	14.52	18.58	15.35	1234.59	117.31	0.79	2.85	0.46	5	49.03
Ơ^2^_REC_	7.16	10.67	4.21	760.34	16.5	0.08	0.62	0.09	1.42	65.06
Residuals	32.32	28.76	4.51	516.29	69.77	2.43	0.55	0.32	1.62	102.33
V_A_	458.18	257.33	166.01	3964.82	1187.94	7.11	0.42	2.48	0.4	180.26
V_D_	14.52	18.58	15.35	1234.59	117.31	0.79	2.85	0.46	5	49.03
h^2^-narrow sense	0.82	0.78	0.76	0.34	0.76	0.78	0.31	0.71	0.35	0.28
H-broad sense	0.84	0.82	0.85	0.39	0.81	0.85	0.85	0.86	0.85	0.44
Predictability ratio (PR)	0.97	0.93	0.92	0.76	0.91	0.9	0.13	0.84	0.07	0.79

Ơ^2^GCA, variance due to general combining ability; Ơ^2^ SCA, variance due to specific combining ability; V_A_, additive variance; V_D_, dominance variance.

### General combining ability

The eight pearl millet parental lines showed different behaviors in this study, carried out in three locations and two rainy seasons (**Table 5**). For grain Fe concentration across sites, only Jirani, MORO, and LCIC 9702 had, in descending order, the positive and significant general combining ability for grain Fe content. On the other hand, for grain Zn concentration, only Jirani and MORO had positive significant GCA. Out of the eight parental lines, three showed negative and significant effects, whereas five exhibited positive and significant effects for flowering time. For plant height, positive significant general combining ability was observed for ZANGO, HKP, ICMV 167006, and ICMV IS 89305. However, significant negative GCA effects were observed for Jirani, MORO, and LCIC 9702. Similar trends were observed for panicle length except for ICMV 167006 and Gamoji, which had respectively negative and positive significant GCA effects. For panicle girth, only the parental lines Gamoji followed by LCIC 9702 had positive GCA. The rest of the parents had either a negative significant or non-significant GCA. Across sites, only ICMV 167006 had significant positive general combining ability effects for grain yield. Concerning panicle compactness, the parental lines ICMV 167006 and MORO had negative significant general combining ability effects. The mean squares of the parents’ LCIC 9702, Gamoji, and HKP were positive and significant for the test weight. For downy mildew incidence, only parent strains ICMV IS 89305 and ZANGO had negative significant GCA effects.

**Table 5:** General combining effects of 8 pearl millet parents over three locations evaluated in two rainy seasons using Griffing's method 1.

	Grain Fe content	Grain Zn content	Flowering time	Plant height	Panicle length	Panicle girth	Grain yield	Compactness of panicle	Thousand seed weight	Downy mildew incidence
Gamoji	-6.51*** (-2.63*)	-2.46* (-3.47**)	12.47*** (7.74***)	1.01^ns^ (-1.7^ns^)	1.5^ns^ (1.39^ns^)	8.89*** (6.75***)	3.00* (-1.44^ns^)	3.68** (9.29***)	1.96^ns^ (2.19*)	-0.7^ns^ (-0.62^ns^)
LCIC 9072	5.84*** (3.95**)	-0.83^ns^ (1.1^ns^)	-16.13*** (-10.07***)	-9.07*** (-6.36***)	-5.06*** (-6.71***)	-0.19ns (3.99**)	-2.76* (0.57^ns^)	1.67ns (3.79**)	3.79** (2.41*)	0.05^ns^ (1.13^ns^)
ICMV IS 89305	-7.61*** (-2.84*)	-5.49*** (-3.08**)	10.94*** (7.44***)	2.68* (2.62*)	4.92** (5.26***)	0.57^ns^ (0.57^ns^)	0.74^ns^ (1.93^ns^)	-0.19ns (-0.58^ns^)	-3.37** (-3.51^ns^)	-4.35** (-4.94**)
ICMV 167006	1.4^ns^ (-0.44^ns^)	-0.95^ns^ (-0.79^ns^)	16.03*** (4.22**)	7.76*** (3.27**)	-4.31** (-6.13***)	-2.84* (-3.76**)	5.98*** (2.59*)	-6.39*** (-14.37***)	-2.16* (-0.71^ns^)	-1.92^ns^ (2.63*)
MORO	8.68*** (6.17***)	7.55*** (7.32***)	-7.07*** (-4.71**)	-1.91^ns^ (-1.6ns)	-7.67*** (-9.74***)	-5.36*** (-5.42***)	-0.17^ns^ (1.54^ns^)	-5.77*** (-11.40***)	-4.39** (-5.15***)	-0.7^ns^ (-0.48^ns^)
Jirani	20.29*** (10.92***)	13.14*** (11.48***)	-31.00*** (-19.14***)	-13.06*** (-8.52***)	-10.06*** (-13.84***)	1.83^ns^ (0.1^ns^)	-7.51*** (-4.34***)	2.75* (8.50***)	0.9ns (1.25^ns^)	7.77*** (2.94*)
HKP	-12.27*** (-8.53***)	-6.46*** (-7.51***)	10.73*** (7.11***)	6.90*** (4.99***)	10.08*** (13.72***)	-2.27* (-1.53^ns^)	2.45* (0.93^ns^)	2.13* (2.40*)	2.09ns (1.83^ns^)	2.31* (3.95**)
ZANGO	-9.83*** (-6.60***)	-4.50** (-5.04***)	4.04** (7.40***)	5.69*** (7.31***)	10.61*** (16.05***)	-0.63^ns^ (-0.69^ns^)	-1.73^ns^ (-1.76^ns^)	2.13* (2.39*)	1.18ns (1.67^ns^)	-2.46* (-4.61**)

ns, non-significant; *, significant at P<0.05; **, significant at P <0.01; ***, significant at P <0.001; The values outside the parentheses are for 2017 rainy season and inside the parentheses are for 2018 rainy season.

### Specific combining ability

Significant positive and negative SCA effects were observed for all the traits of interest among the direct crosses and reciprocal crosses (**Table 6**). For grain Fe concentration, only the hybrids LCIC 9702 × Jirani and MORO × ZANGO exhibited positive significant specific combining ability. As for grain Zn concentration, positive significant SCA effects were observed for the hybrids Gamoji × MORO, LCIC 9702 × Jirani, and ICMV 167006 × Jirani. For grain yield, the specific combining ability (SCA) varied from −4.1 (Jirani × MORO) to 5.1 (LCIC 9702 × ICMV 167006). Positive significant SCA effects for grain yield were observed for seven crosses, and among these crosses, ICMV 167006 was the parent of five hybrids. For flowering time, panicle compactness, and downy mildew incidence, respectively, nine hybrids, six hybrids, and three hybrids had negative significant SCA effects, while the best SCA effects were respectively recorded for the hybrids MORO × Jirani, LCIC 9702 × MORO, and HKP × LCIC 9702. In contrast, for test weight, plant height, panicle length, and panicle girth, positive significant SCA effects were observed for twelve hybrids, seven hybrids, three hybrids, and two hybrids.

**Table 6:** Specific combining effects of 28 pearl millet direct and reciprocal crosses over 3 locations evaluated in two rainy seasons (2017 & 2018) using Griffing's method 1.

	GFe		GZn		FLO		Pht		PL		PG		GY		Pcom		1000sdw		DM	
Direct crosses	2017	2018	2017	2018	2017	2018	2017	2018	2017	2018	2017	2018	2017	2018	2017	2018	2017	2018	2017	2018
Gamoji x LCIC 9072	-0.91	-0.01	-1.65	0.65	1.31	1.23	-0.69	0.47	0.65	0.35	0.35	0.72	1.09	0.63	-2.02*	0.81	-0.69	1.38	-0.45	-1.36
Gamoji x ICMV IS 89305	-0.9	0.5	-0.8	0.3	-0.19	-0.6	1.6	-1.07	0.44	-1.7	-0.31	-0.99	1.86	0.13	-0.86	-0.37	0.87	-0.12	1.2	1.47
Gamoji x ICMV 167006	-0.89	0.53	-0.36	0.86	-2.10*	-2.83**	0.85	-2.89**	0.34	-0.96	0.02	-1.63	1.93	-3.74**	-1.79	-1.78	1.76	0.03	1.96*	-0.24
Gamoji x MORO	-1.45	1.49	0.75	2.84**	0.58	-0.27	1.69	2.67**	0.8	1.37	0.4	-0.17	1.14	3.64**	-0.62	-0.54	0.48	-0.13	-1.96*	-0.83
Gamoji x Jirani	2.23*	-2.58*	1	-1.88	0.54	0.78	1.26	0.21	1	-0.45	0.54	0.91	1.81	1.18	1.29	0.93	0.94	0.28	-1.85	-1.4
Gamoji x HKP	0.09	0.4	-0.79	0.44	0.14	1.14	-0.75	1.31	0.14	-0.15	0	0.11	-1.21	0.42	-0.33	1.8	-1.45	-0.3	0.49	-0.26
Gamoji x ZANGO	0.81	0.56	-0.36	-0.92	-0.53	-1.08	1.67	2.04*	-0.03	1.4	-0.05	-0.52	-0.33	2.24*	0.13	-2.42**	0.54	1.61	0.49	-0.05
LCIC 9072 x ICMV IS 89305	-0.17	-0.25	-0.08	0.76	-1.54	0.1	-0.42	0.7	-0.54	2.46*	-1.25	-0.74	-0.93	0.64	-1.03	0.28	1.6	2.13*	0.01	0.43
LCIC 9072 x ICMV 167006	1.34	-0.85	-0.48	-0.48	-0.4	0.94	2.23*	2.02*	1.16	0.15	-0.73	3.71**	1.09	5.48**	0.36	-2.06*	0.85	1.78	0.42	0.16
LCIC 9072 x MORO	1.29	-1.76	0.64	-2.45**	-3.68**	-1.43	2.87**	0.98	-0.04	-0.01	0.21	-1.02	1.9	-0.07	-1.26	-3.64**	0.24	1.57	-1.06	-0.45
LCIC 9072 x Jirani	0.42	3.32**	1.41	2.15*	-0.69	-2.74**	0.05	-0.77	-0.95	-1.44	2.43*	0.38	0.65	-1.67	1.12	1.07	0.12	-1.53	-0.51	-0.82
LCIC 9072 x HKP	0.47	-1.06	0.76	-0.34	2.09*	0.23	0.93	0.83	1.21	0.11	0	0.67	1.64	1.72	1.35	-1.77	-0.88	0.42	3.46**	2.55**
LCIC 9072 x ZANGO	-0.76	-0.69	-0.1	0.05	-3.02**	-3.11**	-1.36	0.44	-0.4	0.92	0.71	-0.11	-0.39	1.73	0.42	1.51	-0.23	1.18	-0.51	0.61
ICMV IS 89305 x ICMV 167006	0.48	0.03	0.66	-0.25	0.76	-1.27	1.87	0.3	-0.55	-1.3	5.59**	0.42	2.75**	2.44**	1.52	-0.87	0.45	0.55	-0.71	-1.25
ICMV IS 89305 x MORO	-1.38	-0.94	-0.74	-0.66	-1.89	1.45	-0.53	1.35	-0.53	-0.66	-0.45	0.73	-0.2	1.23	0.83	0.33	1.54	0.4	0.18	0.45
ICMV IS 89305 x Jirani	3.29**	-0.19	1.9	-0.13	-0.42	-0.73	-0.84	1.17	-0.94	-0.41	-1.82	-1.02	-1.11	-0.38	-3.30**	-1.48	-1.29	-0.47	-0.13	0.23
ICMV IS 89305 x HKP	0.1	0.57	0.36	0.09	0.71	-0.24	0.7	-0.08	-0.08	-0.34	-1.6	0.38	0.64	0.06	-1.2	-3.42**	-0.63	0.32	-1.63	-2.12*
ICMV IS 89305 x ZANGO	-1.38	0.01	-1.06	-0.98	-0.59	-0.6	-0.97	1.15	5.62**	5.81**	0.99	1.64	0.47	1.61	2.51**	2.21*	0.23	-0.44	-0.07	0.14
ICMV 167006 x MORO	-0.59	-0.76	-1.23	-1.24	3.56**	-2.31**	-3.14**	-0.65	-0.13	0.01	-1.06	0.31	0.47	1.74	1.76	2.21*	0.02	-2.57**	1.11	-0.61
ICMV 167006 x Jirani	1.3	0.82	1.93	0.98	-1.3	1.85	0.32	1.19	0.9	0.89	-1.49	-0.19	-0.14	0.5	-0.97	-1.01	2.09*	2.75**	-1.34	0.62
ICMV 167006 x HKP	-3.09**	-0.16	-2.27*	-2.07*	-1.57	-1.02	1.45	1.04	1.12	1.23	-0.33	-0.21	2.12*	2.78**	-0.74	0.33	-0.17	-0.23	-1.47	2.12*
ICMV 167006 x ZANGO	0.39	-0.86	0.13	-0.14	-3.13**	-0.88	0.63	0.4	-1.18	0.47	-0.75	-0.52	1.09	0.19	-1.67	-1.07	1.94	2.97**	-0.84	-1.27
MORO x Jirani	-0.95	0.05	-1.69	-0.64	-3.06**	-3.13**	-1.3	-0.43	0.53	0.56	-0.17	0.12	-0.3	2.12*	0.65	-0.71	-1.69	-0.33	2.60**	1.74
MORO x HKP	-0.52	-0.89	-0.16	-0.15	-0.42	-0.29	0.92	0.3	-0.89	-0.43	1.75	-0.21	-0.09	-0.02	-1.44	0.16	1.19	0.21	-1.41	0.37
MORO x ZANGO	1.94	1.2	0.77	1.2	-1.59	0.97	0.51	-0.38	0.02	0.3	0.19	0.26	0.22	-2.54**	-0.04	0.16	0.18	1.37	1.67	0.1
Jirani x HKP	-0.02	-0.77	-1.9	-0.27	-0.08	-2.71**	0.59	-0.47	-0.08	0.09	0.38	0.08	-0.09	1.31	0.01	-0.7	3.52**	2.73**	0.24	0.28
Jirani x ZANGO	-5.28**	-2.10*	-3.91**	-1.32	1.41	0.78	1.64	1.22	0.39	0.39	-0.42	0.08	1.37	0.6	0.01	2.57**	2.48**	0.97	0.65	-0.22
HKP x ZANGO	2.63*	0.14	3.03*	-0.27	-0.13	-0.47	0	0.55	0.64	-1.1	-0.02	0.85	1.07	0.69	0.71	0.63	0.88	0.12	-0.36	-0.67
**Reciprocal crosses**																				
LCIC 9072 x Gamoji	-1.92	-0.04	1.57	-0.55	1.47	-1.51	0.29	-0.48	0.83	1.67	-0.41	-0.96	1.23	-0.83	1.01	-0.91	0.63	-0.39	2.26*	-0.95
ICMV IS 89305 x Gamoji	-1.92	0.04	3.36**	0.11	-1.45	-1.5	-0.47	0.14	0.5	0.14	0.33	-0.35	0.82	0	-0.5	-0.91	-1.1	-0.04	0.47	-0.13
ICMV 167006 x Gamoji	-1.6	0.39	-2.02*	-1.9*	1.67	3.22**	0.07	2.21*	-0.67	1.86	3.53**	5.69**	-1.23	1.66	-0.47	0.36	2.22*	-1.26	1.51	-0.49
MORO x Gamoji	0.68	2.53**	0.78	0.11	-0.31	-0.37	-0.83	-0.38	0.5	0	1.79	-0.26	0	-0.41	1.58	-0.04	0.52	-1.09	1.31	-1.3
Jirani x Gamoji	0.61	1.55	-3.36**	-2.41**	-1.25	-1.11	0.71	0.59	0.67	0.28	0.84	1.65	-0.41	-0.83	1.92	1.39	-1.7	-1.36	-0.84	-1.71
HKP x Gamoji	1.12	-1.5	-0.45	-0.88	-0.57	-2.93**	-0.49	-2.31**	0.5	-0.97	-1.54	-0.81	0.41	0.41	-0.48	0.69	0.95	1.56	-0.36	0.58
ZANGO x Gamoji	-0.56	2.44**	-0.78	0.99	1.17	-1.41	1.74	1.83	1.33	0.14	0.35	-2.61**	1.64	1.66	1.59	2.11*	-0.99	1.05	-0.66	0.58
ICMV IS 89305 x LCIC 9702	0.58	-0.65	-0.78	1.43	-0.19	0.78	0.66	0.93	0.67	-0.83	-0.42	-0.91	0	-0.41	0.11	0.82	0.47	0.76	-0.01	-0.36
ICMV 167006 x LCIC 9702	0.49	1.3	2.58**	2.41**	-0.06	-0.01	-0.24	0.67	-0.33	-3.61**	-0.35	0.02	-0.82	0.41	1.2	0.71	0.54	0.49	0.88	0.42
MORO x LCIC 9702	0.36	0.18	1.01	1.98*	-1.2	-0.2	-1.1	-0.34	0	-0.28	-1.82	-1.13	-0.41	0.83	-1.96	-0.69	-0.89	-0.36	0.46	0.59
Jirani x LCIC 9702	-1.51	0.18	2.92**	0.88	-0.62	0	-0.39	0.76	2*	-0.56	1.16	-0.32	1.23	0.04	-0.02	1.13	-1.72	-3.00**	-2.41**	-4.52**
HKP x LCIC 9702	-3.60**	-3.40**	-0.11	-0.11	0.89	-0.01	0.69	0.9	-0.17	-0.28	0.08	1.14	1.23	2.07*	0.48	0.42	-0.09	-0.2	0.05	-0.1
ZANGO x LCIC 9702	0.9	-0.02	1.68	-0.11	0.02	-0.28	0.51	0.52	0	0.14	0.43	-0.18	1.23	0.83	-0.32	-0.31	-0.25	0.23	0.14	-0.08
ICMV 167006 x ICMV IS 89305	-0.17	1.28	-0.22	0.55	1.11	1.07	1	1.59	-4.50**	0.14	1.83	0.92	-2.05*	-1.63	-1.61	0.33	0.2	1.26	0.18	1.35
MORO x ICMV IS 89305	1.7	1.03	2.58**	-0.22	-0.51	-1.86	-0.73	-1.31	-0.33	1.25	1.09	-0.75	1.64	-0.41	-1.85	-2.66**	-0.15	-1.91	-0.05	-2.01*
Jirani x ICMV IS 89305	1.12	0.99	0.22	-0.77	0.75	0.56	0.42	0.45	-0.17	0.7	-0.03	0.89	0	2.07*	-2.71**	-3.50**	1.32	-0.14	0.83	-0.73
HKP x ICMV IS 89305	-0.61	-0.27	0	0	1.27	0.62	0.05	0.72	0.33	1.11	0.37	-0.85	0	-0.83	1.05	0.31	-0.2	-0.84	-0.66	-0.29
ZANGO x ICMV IS 89305	-0.17	-1.23	2.47**	0.66	1.68	2.23*	-4.73**	0.38	1.17	0.56	0.95	1.34	1.64	0.83	2.12*	1.08	-0.08	-0.86	-0.04	-0.92
MORO x ICMV 167006	-0.46	0	2.35*	-1.76	-0.54	-0.27	-0.64	-0.9	-0.67	-0.83	-2.18*	-0.46	1.23	-0.83	-1.26	0.27	-1.31	0.71	-1.03	1.05
Jirani x ICMV 167006	0.71	1.68	-1.79	1.1	-1.34	-0.02	0.27	-0.93	0.33	-0.28	0.49	-0.17	0	-1.24	1.79	1.51	-1.48	-1.61	-1.05	-1.22
HKP x ICMV 167006	0	-1.32	-0.11	1.76	0.23	0.63	-0.22	0.69	0.67	-0.56	-2.06*	0.17	-0.82	-2.07*	-1.03	-0.53	0.76	0.29	-0.39	1.62
ZANGO x ICMV 167006	-0.68	0.16	-0.56	-0.66	0.62	0.63	-0.61	0.03	-1.17	0.56	-0.41	-0.74	-1.64	-0.83	1.09	-0.16	0.74	-2.04*	-0.62	-2.58**
Jirani x MORO	-0.46	0.16	0.9	1.21	-0.89	1.07	-0.64	-0.28	0.33	-0.56	-1.74	-3.42**	0.82	0.83	1.31	0.24	-0.61	1.62	-0.72	2.15*
HKP x MORO	-0.61	-0.81	1.57	0.99	-1.52	-0.4	-0.32	1.62	0	0.28	-0.9	-0.32	1.23	0.41	1	-0.88	-0.86	0.05	-1.23	0.22
ZANGO x MORO	-0.83	-1.68	2.13*	-0.88	-0.34	-0.17	-0.12	1.45	-0.17	0.7	0.1	1.46	0.82	0.41	1.65	0.16	-0.43	-0.4	1.13	-1.52
HKP x Jirani	0.78	-1.23	0	-0.22	-0.27	-1.33	0.15	-2.45**	-0.83	-0.7	-0.75	0.47	-0.41	2.06*	0.25	-1.28	0.24	0.49	-0.76	0.02
ZANGO x Jirani	1.36	-0.38	2.69**	0.77	0.7	0.14	-1.27	-0.1	-0.33	0.14	-0.39	0.7	-2.05*	0.83	2.13*	0.62	-0.87	0.5	0.09	-0.39
ZANGO x HKP	1.82	1.48	0.11	0.44	0.26	-0.31	-0.51	0.31	-0.33	-0.28	2.10*	0.11	0.82	0.41	-1.44	-1.7	-2.83**	-0.35	-1.51	-0.92

*, significant at P<0.05; **, significant at P <0.01.

GFe, Grain iron density in ppm; GZn, Grain zinc density in ppm; FLO, flowering time; Pht, plant height in cm; PL, panicle length in cm; PG, panicle girth in cm; GY, grain yield (t/ha); Pcom, panicle compactness (score); 1000sdw, thousand seed weight in g and DM, downy mildew incidence in percentage.

### Heterosis

For grain Fe and Zn concentration assessed across environments, no positive significant mid-parent heterosis or better-parent heterosis was observed for these traits among the 56 hybrids (**Supplementary Table 1**). By contrast, grain yield showed the most heterosis and heterobeltiosis. For grain yield, 22 hybrids had positive significant mid-parent heterosis that ranged from 103.9 to 284.4, whereas for better parent heterosis, nine hybrids had significant estimates that varied from 113.3 to 236.5. The hybrids resulting from the direct and reciprocal cross between the parental lines ICMV 167006 and LCIC 9702 had the highest positive and significant heterosis and heterobeltiosis.

### Interrelation among grain iron and zinc concentration and agronomic traits

The interrelation between grain Fe concentration and grain Zn concentration across environments was the most positive and highly significant correlation (P <0.01) among parental lines and hybrids, with a coefficient of 0.63 and 0.81 (**Table 7**). For parents and hybrids, grain Fe concentration displayed a low to moderately significant and negative correlation with flowering time, plant height, and panicle length. Only hybrids showed a low significant and negative correlation between grain Fe content and grain yield. The grain Zn concentration was significantly negatively correlated with plant height (r = -0.14**, -0.42** for rainy season 2017 and 2018, respectively), panicle length (r = -0.10*, -0.37**), and panicle compactness (r = −0.32**).

**Table 7:** Correlation among grain iron density (GFe), grain zinc density (GZn), and selected agronomic traits in hybrids of pearl millet from a full 8x8 diallel study pooled across 3 locations during 2 rainy seasons.

	GFe	GZn	FLO	Pht	PL	PG	GY	Pcom	1000sdw	DM
GFe	-
GZn	0.63** (0.81**)	–								
FLO	-0.44** (-0.01)	-0.18** (0.08)	–							
Pht	-0.28** (-0.37**)	-0.14** (-0.42**)	0.36** (-0.23**)	–						
PL	-0.36** (-0.42**)	-0.10* (-0.37**)	0.31** (-0.03)	0.10* (0.51**)	–					
PG	0.04 (-0.06)	0.15** (-0.07)	0.06 (-0.14**)	0 (0.06)	0.12** (0.25**)	–				
GY	0.08 (-0.26**)	0.43** (-0.35**)	0.20** (-0.55**)	0.23** (0.59**)	0.23** (0.18**)	0.21** (0.10*)	–			
Pcom	-0.13** (-0.03)	-0.32**	-0.08 (0.01)	-0.07 (-0.11**)	-0.01 (0.08)	0.01 (0.32**)	-0.40** (-0.13**)	–		
1000sdw	0.09* (-0.13**)	0.19** (-0.19**)	-0.03 (-0.60**)	-0.01 (0.28**)	0.12** (0.24**)	0.24** (0.18**)	0.31** (0.38**)	-0.13** (0.04)	–	
DM	0.06 (0.01)	-0.15** (0)	-0.17** (-0.07)	-0.02 (-0.02)	-0.15** (-0.04)	-0.14** (-0.02)	-0.26** (-0.07)	0.16** (-0.04)	-0.13** (0)	–

*, ** r value significant at P<0.05 and P <0.01, respectively; GFe, Grain iron density; GZn, Grain zinc density; FLO, flowering time; Pht, plant height; PL, panicle length; PG, panicle girth; GY, grain yield; Pcom, panicle compactness; 1000sdw, thousand seed weight, and DM, downy mildew incidence; the values out side the parentheses are for rainy season 2017 and inside are for rainy season 2018.

A low positive and highly significant correlation (r = 0.31**) was found for panicle length and flowering time during the rainy season 2017. Plant height was positively significantly correlated with panicle length (r = 0.10*, 0.51**) and grain yield (r = 0.23**, 0.59**). The panicle length had a low positive significant correlation with panicle girth (r = 0.12**, 0.25**), grain yield (r = 0.23**, 0.18**), and test weight (r =0.12**, 0.24**). For panicle girth, a positive correlation with grain yield (r = 0.21**, 0.10*), panicle compactness (r = 0.32**, during rainy season 2018), and test weight (r = 0.24**, 0.18**) was recorded. A significant positive correlation (r = 0.31**, 0.38**) was registered for grain yield and test weight.

### Stability analysis for grain yield

**Figure 2:**
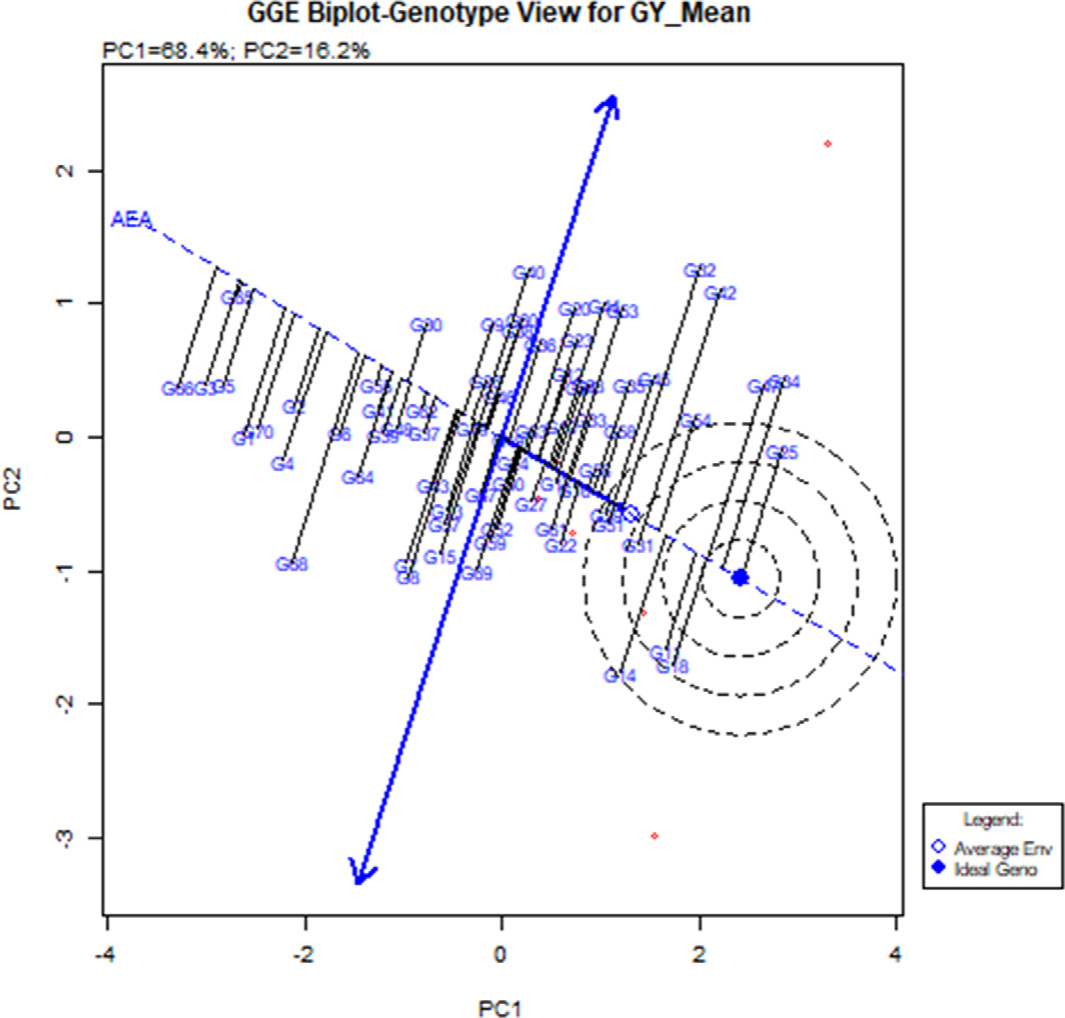
Polygon views of “which-won-where” of the GGE biplot of grain yield of 56 hybrids, 8.

Stability analysis for grain yield, with respect to grain yield, the GGE biplot analysis of 56 hybrids (direct and reciprocal crosses), eight parents, and six checks across six environments revealed that the principal component axis 1 (PC1) explained 68.4% of total variation and the principal component axis 2 (PC2) explained 16.2% of the total variation, with both PC1 and PC2 describing 84.6% of the total variation for grain yield (**Figure 2** and **Table 8**). The most desirable genotypes for grain yield in descending order were G25 (ICMV IS 89305 × ICMV 167006), G34 (ICMV 167006 × LCIC 9702), G47 (LCIC 9702 × ICMV 167006), G18 (HKP × ICMV 167006), G11 (Gamoji × ICMV167006), G14 (Gamoji × MORO), G54 (MORO × ICMV 167006), G31 (ICMV 167006 × HKP), G42 (Jirani × MORO), G32 (ICMV 167006 × ICMV IS 89305), and G51 (MORO × Gamoji). Among these genotypes, G31 (ICMV 167006 × HKP) was the most stable.

**Table 8:** Information on pearl millet genotypes and the environments for the **Figure 2**, **3**.

Genotype	Code	Genotype	Code	Genotype	Code	Environment	Code
CHAKTI	G1	ICMV IS 89305 x LCIC 9702	G26	MORO x HKP	G51	Gaya Rainy Season 2017	E1
Gamoji	G2	ICMV IS 89305 x MORO	G27	MORO x ICMV IS 89305	G52	Gaya Rainy Season 2018	E2
GB 8735	G3	ICMV IS 89305 x ZANGO	G28	MORO x ICMV 167006	G53	Maradi Rainy Season 2017	E3
HKP	G4	ICMV 167006 x Gamoji	G29	MORO x Jirani	G54	Maradi Rainy Season 2018	E4
ICMV IS 89305	G5	ICMV 167006 x HKP	G30	MORO x LCIC 9702	G55	Sadore Rainy Season 2017	E5
ICMV IS 92222	G6	ICMV 167006 x ICMV IS 89305	G31	MORO x ZANGO	G56	Sadore Rainy Season 2018	E6
ICMV IS 99001	G7	ICMV 167006 x Jirani	G32	ZANGO x Gamoji	G57		
Gamoji x HKP	G8	ICMV 167006 x LCIC 9702	G33	ZANGO x HKP	G58		
Gamoji x ICMV IS 89305	G9	ICMV 167006 x MORO	G34	ZANGO x ICMV IS 89305	G59		
Gamoji x ICMV 167006	G10	ICMV 167006 x ZANGO	G35	ZANGO x ICMV 167006	G60		
Gamoji x Jirani	G11	Jirani x Gamoji	G36	ZANGO x Jirani	G61		
Gamoji x LCIC 9702	G12	Jirani x HKP	G37	ZANGO x LCIC 9702	G62		
Gamoji x MORO	G13	Jirani x ICMV IS 89305	G38	ZANGO x MORO	G63		
Gamoji x ZANGO	G14	Jirani x ICMV 167006	G39	ICMV 167006	G64		
HKP x Gamoji	G15	Jirani x LCIC 9702	G40	Jirani	G65		
HKP x ICMV IS 89305	G16	Jirani x MORO	G41	LCIC 9702	G66		
HKP x ICMV 167006	G17	Jirani x ZANGO	G42	ICMV 167005	G67		
HKP x Jirani	G18	LCIC 9702 x Gamoji	G43	MORO	G68		
HKP x LCIC 9702	G19	LCIC 9702 x HKP	G44	SOSAT-C88	G69		
HKP x MORO	G20	LCIC 9702 x ICMV IS 89305	G45	ZANGO	G70		
HKP x ZANGO	G21	LCIC 9702 x ICMV 167006	G46				
ICMV IS 89305 x Gamoji	G22	LCIC 9702 x Jirani	G47				
ICMV IS 89305 x HKP	G23	LCIC 9702 x MORO	G48				
ICMV IS 89305 x ICMV 167006	G24	LCIC 9702 x ZANGO	G49				
ICMV IS 89305 x Jirani	G25	MORO x Gamoji	G50				

**Figure 3:**
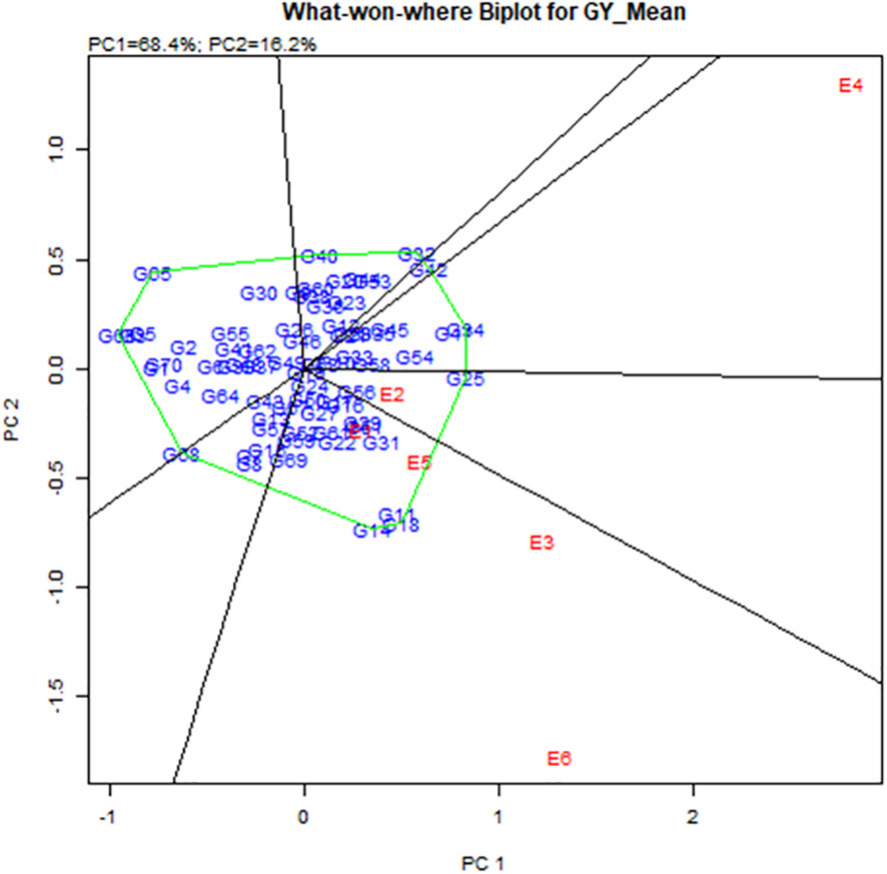
Polygon views of “which-won-where” of the GGE biplot of grain yield of 56 hybrids, 8.

The hybrids G11, G18, and G14 were the vertex genotypes in the sector that included the environments E1, E3, E5, and E6 (respectively Gaya Rainy Season 2017, Maradi Rainy Season 2017, Sadore Rainy Season 2017, and Sadore Rainy Season 2018). The genotype G25 was the vertex of the sector containing E2 (Gaya Rainy Season 2018), and the genotypes (G34 and G47) were the vertex genotypes in the sector that contained the environment E4 or Maradi Rainy Season 2018 (**Figure 3**).

## Discussion 

### Parents and hybrids performance *per se*

Based on the mean performance across all six environments, high variability of grain Fe and Zn concentration among the parents and hybrids was found in the present study. This suggests the possibility of improving these traits through plant breeding. Similar findings were reported by Rai et al. (2013); Rai et al. (2014); Rai et al. (2015), that there is a large variability in grain Fe and Zn content in pearl millet germplasm and breeding materials. The varieties with high grain Fe and Zn concentration (Jirani, LCIC 9702, CHAKTI, GB 8735) are all of the Iniari parentage. The Iniari germplasm from Togo, Benin, Ghana, and Burkina Faso has been extensively used in pearl millet programs due to its early maturity and large seed size, and it has been found to be the best source of Fe and Zn content in pearl millet (Andrews and Kumar, 1996; Rai et al., 2008; Velu et al., 2011; Rai et al., 2012; Govindaraj et al., 2019).

### Estimates of variance and genetic variance components

For grain Fe and Zn concentration, the relatively high proportions of GCA over SCA, the high ratio of additive and dominance variance, and the predictability ratio close to unity (0.97 for iron and 0.93 for zinc) suggest that these traits are predominantly under the control of additive gene action. This is in line with previous studies that reported these traits being under additive gene action in pearl millet (Velu, 2006; Rai et al., 2007; Govindaraj et al., 2013; Kanatti et al., 2014; Kanatti et al., 2016; Pujar et al., 2022). With respect to the agronomic traits, flowering time, plant height, panicle length, panicle girth, panicle compactness, and downy mildew incidence had a high predictability ratio, implying that these traits are under additive genetic control. Several studies have reported these traits being under additive gene action (Izge et al., 2007; Kanfany et al., 2018; Subbulakshmi et al., 2019). However, grain yield and test weights had very low predictability ratios of 0.13 and 0.07, respectively, indicating non-additive gene action to be predominant in the inheritance of these traits.

A high narrow sense heritability was found for grain Fe concentration (82%) and grain zinc concentration (78%) across the environments, which indicate that both grain Fe and Zn concentration are highly heritable. Previous studies on pearl millet have reported a high narrow-sense heritability for grain Fe and Zn concentrations ranging from 65% to 86% for both micronutrients (Kanatti et al., 2016). To sum up, these findings might imply that genetic improvement for grain Fe and Zn concentrations can be effective in pearl millet.

The reciprocal effects were significant for grain zinc content, grain yield, flowering time, plant height, test weight, and downy mildew incidence, which suggests that the choice of female or male parent is critical to improving these traits in order to avoid missing potential good hybrids. For instance, the top cross hybrid Jirani × LCIC 9702 that had the highest grain Fe and Zn concentration was a reciprocal cross. The partitioning of the reciprocal effects into maternal and non-maternal effects for grain Fe and Zn content showed that there was a difference between maternal effects and non-maternal effects (significant only for grain zinc concentration) and the mean squares of maternal effects were higher than non-maternal effects for both grain Fe and Zn concentration, suggesting that maternal effects may influence to a certain extent the inheritance of these traits. In a study on maize, Chakraborti et al. (2011) reported significant reciprocal differences in the diallel crosses of quality protein maize for kernel Fe and Zn concentrations. For grain yield, only the non-maternal effects were significant in our experiment. These effects are mainly caused by the specific interactions between cytoplasm and nucleus genes (Zhang and Kang, 1997; Mahgoub, 2011).

### General combining ability

The parental lines Jirani, MORO, and LCIC 9702 had, in descending order, superior positive GCA effects for grain Fe and Zn concentration, except LCIC 9702 had non-significant GCA effects for grain zinc concentration. Therefore, these parents contributed to higher grain Fe and Zn concentrations in their hybrids, making them the best general combiners to use in a biofortification program. For the agronomic traits, the best combiners were identified for plant height (ZANGO, HKP, ICMV 167006, and ICMV IS 89305), for panicle length (ZANGO, HKP, and ICMV IS 89305), for panicle girth (Gamoji and LCIC 9702), for grain yield (ICMV 167006), and for test weight (LCIC 9702, Gamoji, and HKP). For disease resistance, flowering time, and panicle compactness, the negative GCA effects are desired, thus the best combiners for a flowering time were Jirani, LCIC 9702, and MORO; for panicle compactness, they were ICMV 167006 and MORO; and for downy mildew incidence, ICMV IS 89305 and ZANGO. All these combiners are likely to contribute to favorable alleles in recurrent selection programs and can also be used as parents to develop synthetic populations.

### Specific combining ability

For grain Fe and Zn concentration, respectively, only two crosses, LCIC 9702 × Jirani and MORO × ZANGO, and three crosses, Gamoji × MORO, LCIC 9702 × Jirani, and ICMV 167006 × Jirani displayed positive significant SCA effects. These crosses originated from high and high or low and high GCA effects parents and had good performance per se across sites. This is in line with Izge et al. (2007), who reported that hybrids that generally perform well exhibit significant SCA effects.

### Heterosis

The absence of positive significant mid-parent heterosis and better parent heterosis among the 56 hybrids suggests that heterosis offers limited opportunities for the improvement of these micronutrients in pearl millet. Similar findings were reported in pearl millet (Govindaraj, 2011; Velu et al., 2011; Manwaring et al., 2016). Kanatti et al. (2014) showed that in order to efficiently breed pearl millet hybrids rich in iron and zinc, these micronutrients should first be incorporated into both parents. The high positive heterosis and heterobeltiosis recorded for many hybrids for grain yield and test weight suggest that heterosis can be exploited to breed for these traits in pearl millet hybrids.

### Interrelation among grain iron and zinc concentration and agronomic traits

Among the parents and the hybrids, a strong positive correlation between grain Fe and Zn concentration was observed across environments, with respective values of 0.74 and 0.71 (P <0.001). These findings may suggest that grain Fe and Zn concentrations can be selected simultaneously in a single improved variety in biofortification breeding programs. These results are in line with previous studies that demonstrated a positive significant correlation between these traits (Velu et al., 2007; Gupta et al., 2009; Govindaraj, 2011; Kanatti et al., 2014). Both grain Fe and Zn concentrations were not correlated either with grain yield, panicle girth, and test weight or with downy mildew incidence in the parental lines. This means that parental lines having high grain Fe and Zn content could be developed without compromising on these traits. In contrast, in hybrids, grain iron had a highly significant and low negative correlation with grain yield. Many studies have reported that there was a negative relationship between grain yield and micronutrients (Banziger and Long, 2000; Reddy et al., 2005; Garvin et al., 2006; Jiang et al., 2007; Morgounov et al., 2007; Shi et al., 2008; Govindaraj et al., 2009; Zhao et al., 2009; Prasad, 2010; Anusha et al., 2021; Srungarapu et al., 2022). The decline in micronutrient concentration as grain yield increases is probably due to a dilution effect. Therefore, taking into account the weak negative correlation between grain yield and grain Fe content, it is possible to select high-yielding hybrids with high grain Fe content by selecting from larger segregating populations and progenies. Similarly, grain Fe and Zn content had a weak to moderate negative correlation with plant height and panicle length. These findings might also be partially explained by the effect of the sink. The more the plant and panicle size is increased, the more the dilution effect of minerals in the grain is important. However, these findings do not imply that by reducing plant height and panicle length, the grain will be richer in iron and zinc. The plant height, panicle length, panicle girth, and test weight had low positive correlations with grain yield across sites. This could be the effect of increased seed production that resulted in high grain yield. This is in line with previous reports on pearl millet (Govindaraj et al., 2009; Patil et al., 2018). Both seed set and long panicle were found to be correlated with the yield per unit area, which suggests that farmers will likely adopt an early variety with a long panicle and a good seed set for better yield (Gupta et al., 2015; Ausiku et al., 2020).

### Stability analysis for grain yield

In this study, the best yielding stable hybrid across environments was the hybrid ICMV 167006 × HKP (average 2.3 t/ha). Among the parental lines, ICMV 167006 was the only one with positive significant GCA effects for grain yield. Moreover, in a study conducted by Bashir et al. (2014), they found that B9_Tabi (parent of ICMV 167006) had a high tillering capacity and was the most stable genotype under severe drought. Many authors reported that the high tillering capacity of pearl millet varieties increases their plasticity, under severe drought the secondary tillers are able to compensate for the losses of the main tillers (Vadez et al., 2012; Haussmann et al., 2012; Srivastava et al., 2022). The most stable high-yielding (1.9 t/ha) hybrid with high grain iron (48.9 ppm) and zinc (42.3 ppm) content was ICMV 167006 × Jirani (G33). This genotype should be extensively evaluated and promoted for commercialization to contribute to improved micronutrient nutrition and pearl millet production and productivity in Sub-Saharan Africa. The hybrids G11, G18, and G14 were the vertex genotypes in the sector that contained the environments E1, E3, E5, and E6, which means that they were the highest-yielding genotypes (winning genotypes) for this set of environments.

## Conclusions

Based on the overall performance of the genotypes across environments, the most effective combiners (GCA) for grain Fe concentration (Jirani, MORO, and LCIC 9702) and for grain zinc concentration (Jirani and MORO) could be used to develop hybrids, synthetic varieties, and open-pollinated varieties by the National Agricultural Research Systems (NARS) and international research institutes. The absence of positive significant mid-parent and better parent heterosis for grain Fe and Zn in the hybrids generated from this study, indicates that heterosis offers limited opportunities for the improvement of these micronutrients in pearl millet. Therefore, to breed for these micronutrients, they should first be incorporated into both parents. However, for grain yield and test weight, the high positive heterosis and heterobeltiosis suggest that heterosis can be exploited to breed for these traits in pearl millet hybrids. This study revealed that grain Fe and Zn concentration, flowering time, plant height, panicle length, panicle girth, panicle compactness, and downy mildew incidence are predominantly under additive gene actions, while grain yield and test weight are predominantly under non-additive gene actions. As a result, the additive nature of the former traits provides scope for their improvement under recurrent selection, while the non-additive nature of grain yield and test weight justifies the initiation of a hybrid program in order to improve these traits by crossing progenitor combinations with superior SCA. The significance of reciprocal effects for grain zinc content, grain yield, flowering time, plant height, test weight, and downy mildew incidence indicated that the choice of which parent is female or male is critical in hybrid production when these traits are considered. In both parental lines and hybrids, a highly positive correlation was found between grain Fe concentration and grain Zn concentration. This suggests that these traits may be improved at the same time in a variety. The GGE biplot allowed the identification of the highest-yielding and most stable hybrid across environments, which was the hybrid ICMV 167006 × HKP (2.3 t/ha).

## Data availability statement

The original contributions presented in the study are included in the article/**Supplementary Material**. Further inquiries can be directed to the corresponding author.

## Author contributions

PG conceived the concept and provided the materials, helped in designing of the diallel, hybrid trial, data curation, and drafting the manuscript. BG conducted the research and statistical data analysis, and drafted the manuscript. IA conducted major part of Maradi location trials. RM helped in data analysis, edited the manuscript with critical suggestions. DD, MG and PT edited and made minor corrections and contributed for manuscript editions. All authors contributed to the article and approved the submitted version.
