# Supplementary material for: Combining ability studies of grain Fe and Zn contents of pearl millet (Pennisetum glaucum L.) in West Africa
Source: Front Plant Sci. 2023 Jan 6;13:1027279. doi: 10.3389/fpls.2022.1027279 (PMC9854276; doi:10.3389/fpls.2022.1027279)
Supplement: Supplementary file 1 [file Table_1.docx]

Supplementary Table 1. Heterosis over mid parent (Ht) and better parent (Hbt) of direct and reciprocal crosses hybrids in an 8*8 full diallel in three locations (pooled) during rainy season 2017 and rainy season 2018

| **CROSS** | **Gfe** | | | | **GZn** | | | | **FLO** | | | | **Pht** | | | | **PL** | | | | **PG** | | | | **GY** | | | | **Pcom** | | | | **1000sdw** | | | | **DM** | | | |
| --- | --- | --- | --- | --- | --- | --- | --- | --- | --- | --- | --- | --- | --- | --- | --- | --- | --- | --- | --- | --- | --- | --- | --- | --- | --- | --- | --- | --- | --- | --- | --- | --- | --- | --- | --- | --- | --- | --- | --- | --- |
|  | 2017 | | 2018 | | 2017 | | 2018 | | 2017 | | 2018 | | 2017 | | 2018 | | 2017 | | 2018 | | 2017 | | 2018 | | 2017 | | 2018 | | 2017 | | 2018 | | 2017 | | 2018 | | 2017 | | 2018 | |
|  | Ht | Hbt | Ht | Hbt | Ht | Hbt | Ht | Hbt | Ht | Hbt | Ht | Hbt | Ht | Hbt | Ht | Hbt | Ht | Hbt | Ht | Hbt | Ht | Hbt | Ht | Hbt | Ht | Hbt | Ht | Hbt | Ht | Hbt | Ht | Hbt | Ht | Hbt | Ht | Hbt | Ht | Hbt | Ht | Hbt |
| Gamoji x LCIC9702 | 0 | -6 | -2 | -12 | 0 | -2 | 3 | -5 | 0 | 7* | -2 | 2 | 15 | 7 | 7 | -1 | 20 | 11 | 4 | -12 | 9 | -3 | 14 | 0 | 88 | 55 | 161 | 97 | -16 | -5 | -12 | -8 | 5 | 2 | 14 | 8 | -63 | -57 | -34 | -18 |
| Gamoji x ICMV IS 89305 | -6 | -8 | 3 | 1 | -4 | -10 | 3 | -3 | 1 | 2 | -2 | -2 | 8 | 2 | 1 | -2 | 18 | 11 | 1 | -1 | 4 | -6 | -5 | -16 | 77* | 67 | 86 | 67 | -14 | 0 | -13 | 0 | 7 | 2 | 4 | -1 | -58 | -40 | -3 | 100 |
| Gamoji x ICMV 167006 | 1 | -5 | -4 | -9 | -2 | -2 | 1 | -9 | -5 | -2 | -6* | -6* | 18* | 10 | 7 | 1 | 19 | 9 | 7 | -5 | 1 | -12 | 3 | -17 | 165** | 153** | 239** | 176* | -35 | -8 | -16 | 45 | 15 | 5 | 12 | 5 | -10 | -8 | -17 | -16 |
| Gamoji x MORO | -5 | -15* | 0 | -12 | 1 | -8 | 6 | -10 | -1 | 2 | -3 | 0 | 12 | 8 | 14 | 12 | 9 | -1 | 6 | -12 | 7 | -10 | -4 | -21 | 87* | 79 | 164* | 148* | -17 | 25 | -16 | 45 | 11 | 6 | 4 | 0 | -23 | -2 | 18 | 62 |
| Gamoji x Jirani | 1 | 22* | -13 | -26** | -4 | -15 | -9 | -25** | -4 | 9* | -4 | 2 | 10 | 1 | 4 | -2 | 28 | 5 | 0 | -20 | 5 | -1 | 2 | -9 | 103* | 59 | 166* | 130 | -9 | 0 | -2 | 4 | 19* | 12 | 14 | 11 | -20 | 52 | -10 | -8 |
| Gamoji x HKP | 1 | -5 | 6 | 3 | -8 | -14 | 3 | 0 | 0 | 1 | -2 | -1 | 8 | 1 | 3 | -2 | 11 | -6 | -10 | -21* | 4 | -8 | -4 | -19 | 23 | 13 | 113 | 99 | -14 | -5 | 0 | 9 | 1 | 1 | 9 | 8 | 27 | 76 | -31 | -23 |
| Gamoji x ZANGO | -4 | -6 | 5 | 4 | -9 | -12 | -1 | -7 | -4 | -4 | -2 | -1 | 16* | 7 | 11 | 6 | 31 | 16 | 26* | 20 | 8 | -3 | -1 | -16 | 60 | 53 | 78 | 74 | 5 | 31 | 2 | 37 | 16 | 11 | 25** | 17 | 13 | 133 | 12 | 82 |
| LCIC9702 x ICMVIS 89305 | 3 | -6 | -1 | -9 | 1 | -4 | 0 | -3 | -5* | 1 | -1 | 3 | 5 | -8 | 15 | 4 | 15 | -1 | 30* | 11 | 4 | 1 | 1 | 0 | 37 | 8 | 153* | 77 | -14 | -11 | -17 | -10 | 9 | 0 | 22* | 22 | 33 | 60 | -2 | 53 |
| LCIC9702 x ICMV 167006 | 5 | 4 | -5 | -9 | 0 | -3 | -3 | -5 | -2 | 7* | -1 | 3 | 15 | 0 | 12 | 0 | 16 | 16 | 12 | 5 | 2 | -1 | 11 | 1 | 116* | 85 | 514** | 457** | -12 | 8 | -33 | 9 | 16 | 2 | 25* | 24* | 39 | 59 | 30 | 62 |
| LCIC9702 x MORO | 1 | -4 | -10 | -12 | 2 | -9 | -6 | -13 | -7* | -4 | -3 | -2 | 10 | 0 | 13 | 7 | -6 | -8 | 8 | 5 | 6 | 0 | 2 | -6 | 52 | 21 | 146 | 78 | -16 | 8 | -33 | 9 | -1 | -9 | 13 | 11 | 24 | 33 | 14 | 25 |
| LCIC9702 x Jirani | 0 | -10 | -4 | -10 | -4 | -17* | -9 | -19 | -2 | 4 | -6* | -3 | 6 | 4 | 7 | 6 | -3 | -15 | 3 | -4 | 19 | 12 | 8 | 7 | 106 | 93 | 131 | 96 | 8 | 11 | -2 | 0 | 9 | 0 | 15 | 12 | -26 | -54 | 2 | 22 |
| LCIC9702 x HKP | 2 | -10 | -9 | -20 | 2 | -2 | -5 | -10 | -1 | 5 | -3 | 1 | 14 | 0 | 12 | 0 | 18 | -7 | 8 | -19 | 3 | 3 | 14 | 8 | 78 | 37 | 315** | 229* | 8 | 11 | -13 | -9 | 3 | 0 | 16 | 11 | -6 | 59 | 7 | 16 |
| LCIC9702 x ZANGO | -2 | -11 | -6 | -16 | -1 | -2 | -3 | -6 | -6* | 0 | -6* | -1 | 4 | -10 | 13 | 1 | 18 | -3 | 28* | 4 | 7 | 6 | 12 | 8 | 64 | 40 | 222* | 146 | 14 | 25 | 12 | 44 | 7 | -1 | 24* | 21 | 89 | 214 | 10 | 40 |
| ICMVIS 89305xICMV 167006 | 0 | -8 | 2 | -2 | 1 | -7 | -1 | -6 | -2 | -1 | -3 | -3 | 13 | 12 | 9 | 6 | 18 | 3 | 11 | 0 | 7 | 1 | 8 | -3 | 127** | 105* | 236** | 151* | -16 | 0 | -43 | -18 | 10 | 5 | 14 | 12 | -71 | -60 | -7 | 96 |
| ICMVIS 89305 x MORO | -5 | -17* | -12 | -22* | -4 | -17 | -11 | -20* | -3 | 1 | -2 | 1 | 0 | -3 | 7 | 3 | 1 | -14 | 2 | -15 | 1 | 10 | 11 | 1 | 45 | 43 | 106 | 95 | 13 | 42 | -19 | 18 | 5 | 5 | -3 | -4 | 97 | 120 | 49 | 106 |
| ICMVIS 89305 x Jirani | 10 | -8 | -3 | -17 | 6 | -12 | -5 | -18 | -3 | 11** | -4 | 2 | 4 | -10 | 12 | 3 | 11 | -14 | 9 | -12 | -6 | -10 | 1 | 0 | 23 | -8 | 108 | 64 | -26 | -22 | -2 | 5 | 0 | 0 | -3 | -5 | 6 | 234 | 25 | 147 |
| ICMV IS 89305 x HKP | -1 | -5 | -5 | -9 | -2 | -3 | -6 | -9 | 0 | 1 | -2 | -2 | 11 | 11 | 9 | 7 | 12 | 0 | 7 | -9 | -2 | -4 | 12 | 5 | 48 | 43 | 103 | 71 | -16 | -11 | -36* | -33 | 8 | 2 | 9 | 5 | -78 | -51 | -37 | 10 |
| ICMV IS 89305 x ZANGO | -6 | -6 | -7 | -9 | -6 | -9 | -12 | -12 | -2 | -2 | -2 | -1 | 6 | 4 | 19* | 16 | 22 | 15 | 43** | 34* | 12 | 11 | 15 | 9 | 57 | 42 | 150* | 119 | 29 | 37 | 19 | 37 | 18* | 18 | 17 | 14 | -21 | 5 | -76 | -71 |
| ICMV 167006 x MORO | -7 | -12 | -4 | -11 | -10 | -17 | -5 | -11 | 1 | 7* | -7** | -5 | -3 | -7 | 4 | -3 | -1 | -3 | -1 | -9 | -3 | -6 | 2 | 0 | 49 | 36 | 206* | 137* | 20 | 25 | -18 | -18 | 8 | 4 | 2 | 0 | 25 | 53 | -7 | 30 |
| ICMV 167006 x Jirani | -1 | -11 | -6 | -16 | -2 | -13 | -6 | -14 | -6* | 10** | -2 | 5 | 5 | -10 | 7 | -4 | 20 | 5 | -1 | -12 | -3 | -12 | 3 | -7 | 96 | 59 | 203* | 181 | -15 | 8 | -26 | 18 | 29** | 24* | 26** | 22* | -17 | 63 | 33 | 39 |
| ICMV 167006 x HKP | -7 | -17* | -5 | -13 | -12 | -18 | -8 | -15 | -2 | 1 | -2 | -2 | 13 | 12 | 9 | 8 | 14 | -10 | 7 | -16 | 5 | 1 | 5 | 0 | 71 | 50 | 302** | 246** | -21 | 0 | -35 | 0 | 9 | -1 | 9 | 4 | -46 | -22 | 21 | 38 |
| ICMV 167006 x ZANGO | 0 | -8 | -16 | -21* | -6 | -10 | -17 | -20* | -7** | -5 | -5 | -4 | 10 | 9 | 10 | 9 | 8 | -11 | 19 | 1 | -4 | -9 | 10 | 3 | 89* | 88 | 162 | 117 | -24 | -15 | -19 | 0 | 27** | 21* | 27** | 26* | -67 | -33 | -35 | 7 |
| MORO x Jirani | -3 | -8 | 1 | -3 | -9 | -13 | 2 | -2 | -6* | 3 | -6* | -2 | -3 | -13 | 10 | 5 | 1 | -10 | 4 | -1 | 1 | -10 | -1 | -10 | 5 | -20 | 68 | 38 | 6 | 42 | -3 | 55 | 10 | 10 | 7 | 5 | 39 | 270 | 43 | 90 |
| MORO x HKP | -5 | -20** | -8 | -21* | -8 | -20* | -5 | -17 | -1 | 1 | -2 | 0 | 4 | -1 | 8 | 2 | -2 | -24 | 6 | -21* | 9 | 3 | 5 | 2 | 25 | 19 | 124 | 98 | -6 | 25 | -12 | 36 | 11 | 6 | 3 | 0 | -43 | 7 | 18 | 43 |
| MORO x ZANGO | 0 | -13 | -3 | -15 | 1 | -10 | -6 | -16 | -5 | -1 | -3 | 0 | 4 | -1 | 10 | 3 | 13 | -9 | 27 | 2 | 3 | -4 | 9 | 4 | 42 | 30 | 85 | 71 | 14 | 33 | 11 | 36 | 16 | 16 | 16 | 13 | 103 | 210 | -54 | -47 |
| Jirani x HKP | 1 | -18** | -5 | -22* | -10 | -25** | -6 | -20* | -1 | 11** | -6* | 1 | 8 | -7 | 2 | -8 | 9 | -22 | -12 | -36** | -3 | -9 | 1 | -4 | 29 | -5 | 181* | 159 | -10 | -10 | 2 | 4 | 20* | 14 | 14 | 11 | 7 | 38 | 3 | 12 |
| Jirani x ZANGO | -13 | -27** | -9 | -23* | -13 | -26** | -10 | -22* | -1 | 13** | -3 | 5 | 11 | -5 | 13 | 2 | 12 | -17 | 18 | -9 | -3 | -8 | 6 | 1 | 59 | 28 | 131 | 105 | -11 | 0 | 30 | 62* | 29** | 28** | 22* | 17* | 45 | 610 | -21 | 24 |
| HKP x ZANGO | -4 | -8 | -7 | -10 | 0 | -3 | -13 | -15 | -3 | -1 | -3 | -2 | 7 | 6 | 10 | 10 | 16 | 9 | 9 | -1 | 0 | -1 | 11 | 9 | 79* | 57 | 146 | 134 | 11 | 25 | 8 | 31 | 9 | 3 | 10 | 4 | 49 | 390 | 30 | 82 |
| **RECIPROCAL CROSS** | **Gfe** | | | | **GZn** | | | | **FLO** | | | | **Pht** | | | | **PL** | | | | **PG** | | | | **GY** | | | | **Pcom** | | | | **1000sdw** | | | | **DM** | | | |
|  | 2017 | | 2018 | | 2017 | | 2018 | | 2017 | | 2018 | | 2017 | | 2018 | | 2017 | | 2018 | | 2017 | | 2018 | | 2017 | | 2018 | | 2017 | | 2018 | | 2017 | | 2018 | | 2017 | | 2018 | |
|  | Ht | Hbt | Ht | Hbt | Ht | Hbt | Ht | Hbt | Ht | Hbt | Ht | Hbt | Ht | Hbt | Ht | Hbt | Ht | Hbt | Ht | Hbt | Ht | Hbt | Ht | Hbt | Ht | Hbt | Ht | Hbt | Ht | Hbt | Ht | Hbt | Ht | Hbt | Ht | Hbt | Ht | Hbt | Ht | Hbt |
| LCIC9702 x Gamoji | -3 | -9 | 1 | -9 | -15 | -17 | 10 | 1 | -3 | 4 | -1 | 3 | 6 | -1 | 16 | 8 | 16 | 7 | 9 | -8 | 3 | -9 | -1 | -13 | 101* | 65 | 219* | 140 | -30 | -21 | -4 | 0 | 0 | -3 | 19* | 13 | 74 | 104 | -32 | -16 |
| ICMVIS 89305 x Gamoji | 0 | -3 | 3 | 1 | -7 | -13 | 4 | -2 | -4 | -4 | -2 | -2 | 16* | 9 | 9 | 6 | 24 | 16 | 0 | -2 | 1 | -9 | -7 | -17 | 69 | 59 | 100 | 80 | -24 | -11 | -13 | 0 | 10 | 4 | 9 | 4 | 98 | 186 | -5 | 96 |
| ICMV 167006 x Gamoji | -10 | -15 | 6 | 0 | -11 | -12 | 4 | -6 | -1 | 1 | -3 | -3 | 9 | 1 | -1 | -7 | 18 | 8 | -13 | -23 | 7 | -8 | -15 | -31* | 69 | 61 | -80 | -84 | -19 | 15 | -37 | 9 | 18* | 8 | 10 | 3 | 92 | 97 | -29 | -28 |
| MORO x Gamoji | -7 | -17* | 7 | -6 | -7 | -15 | 15 | -2 | -3 | 0 | -3 | -1 | 13 | 10 | 16 | 14 | 21 | 10 | 10 | -9 | 3 | -14 | -4 | -21 | 42 | 36 | 175** | 159* | -17 | 25 | -11 | 55 | 2 | -3 | 4 | 0 | -74 | -67 | -77 | -68 |
| Jirani x Gamoji | 8 | 31** | -4 | -18 | 1 | -11 | 2 | -16 | 2 | 16** | 0 | 6* | 18 | 8 | 10 | 4 | 17 | -5 | -6 | -25 | 0 | -6 | 0 | -12 | 75 | 37 | 79 | 55 | -5 | 5 | 6 | 12 | 8 | 2 | 6 | 3 | -40 | 14 | -57 | -56 |
| HKP x Gamoji | -4 | -10 | -7 | -9 | -6 | -12 | -1 | -4 | 1 | 2 | 0 | 0 | 11 | 3 | 18* | 12 | 16 | -2 | 6 | -7 | 0 | -11 | 5 | -12 | 60 | 46 | 153* | 135 | -18 | -10 | -4 | 4 | 4 | 4 | 5 | 4 | -23 | 6 | 17 | 32 |
| ZANGO x Gamoji | 1 | -1 | -4 | -4 | -4 | -7 | -6 | -11 | -3 | -2 | -4 | -3 | 10 | 2 | 18* | 13 | 11 | -2 | 12 | 7 | -3 | -12 | -2 | -17 | 50 | 44 | 200** | 193* | -15 | 6 | -16 | 12 | 7 | 2 | 12 | 5 | 66 | 243 | -86 | -78 |
| ICMVIS89305 x LCIC9702 | 0 | -8 | -6 | -14 | 1 | -4 | 3 | 0 | -4 | 3 | -4 | 0 | 6 | -7 | 10 | 0 | 6 | -8 | 20 | 3 | -2 | -4 | 10 | 8 | 49 | 18 | 200* | 110 | -14 | -11 | -13 | -5 | 8 | 0 | 17 | 16 | -24 | -9 | 36 | 112 |
| ICMV 167006 x LCIC9702 | 2 | 2 | -8 | -12 | -6 | -9 | -6 | -8 | -6* | 2 | -5* | -1 | 16 | 0 | 13 | 0 | 20 | 20 | 4 | -2 | 5 | 1 | 51** | 38** | 128* | 95 | 513** | 455** | 0 | 23 | -39 | 0 | 9 | -4 | 20* | 19 | 4 | 18 | -15 | 7 |
| MORO x LCIC9702 | 4 | -1 | -8 | -10 | -1 | -11 | -10 | -17 | -9** | -6 | -7** | -5 | 17 | 6 | 15 | 8 | 12 | 9 | 12 | 10 | 6 | 0 | 5 | -3 | 107* | 65 | 209* | 124 | -10 | 17 | -44* | -9 | 10 | 1 | 17 | 16 | -9 | -2 | 6 | 16 |
| Jirani x LCIC9702 | 7 | -3 | 14 | 6 | 9 | -5 | 18* | 5 | -8* | -2 | -7** | -5 | 10 | 9 | 7 | 6 | 4 | -9 | -7 | -13 | 3 | -4 | 13 | 12 | 56 | 46 | 155 | 116 | -8 | -5 | -2 | 0 | 9 | 0 | 8 | 5 | 26 | -20 | -4 | 14 |
| HKP x LCIC9702 | 3 | -9 | -8 | -19 | 1 | -3 | -4 | -9 | 0 | 5 | -3 | 2 | 9 | -5 | 12 | 0 | 10 | -13 | 0 | -24* | 4 | 4 | 17 | 11 | 76 | 35 | 239* | 169 | -8 | -5 | -33* | -30 | 1 | -2 | 14 | 9 | 172* | 361* | 141* | 162* |
| ZANGO x LCIC9702 | -1 | -9 | -8 | -17 | -2 | -3 | -3 | -6 | -9** | -3 | -6* | -1 | 4 | -10 | 15 | 2 | 12 | -8 | 23 | 0 | 7 | 6 | 11 | 7 | 50 | 28 | 233* | 155 | -3 | 6 | 2 | 31 | 9 | 0 | 26* | 23* | -17 | 38 | 12 | 41 |
| ICMV 167006 x ICMVIS 89305 | -1 | -9 | -7 | -11 | -1 | -8 | -10 | -15 | -2 | 0 | -4 | -4 | 8 | 6 | 3 | 1 | 5 | -9 | -4 | -13 | 47** | 39** | 7 | -4 | 80* | 62 | 192* | 118 | 16 | 38 | -19 | 18 | 20* | 14 | 11 | 10 | -57 | -40 | -62 | -20 |
| MORO x ICMV IS 89305 | -4 | -16* | 1 | -10 | -4 | -17 | 2 | -9 | -7** | -4 | -1 | 1 | 3 | -1 | 17* | 13 | 11 | -5 | 15 | -4 | 4 | 13 | -2 | -11 | 19 | 17 | 135* | 122* | -13 | 8 | -12 | 27 | 16 | 16 | 14 | 13 | -82 | -80 | -19 | 12 |
| Jirani x ICMV IS 89305 | 5 | -13* | -3 | -16 | 1 | -16 | -1 | -14 | -3 | 11** | -3 | 3 | 0 | -14 | 8 | 0 | 5 | -18 | 4 | -16 | -5 | -9 | -6 | -7 | 24 | -7 | 67 | 32 | -26 | -22 | -24 | -19 | 17 | 16 | 19 | 17 | -35 | 103 | -20 | 57 |
| HKP x ICMV IS 89305 | 0 | -3 | 2 | -3 | 2 | 2 | -4 | -7 | 0 | 1 | -2 | -2 | 4 | 5 | 6 | 4 | 12 | 0 | 1 | -13 | -5 | -7 | 1 | -5 | 40 | 35 | 140* | 102 | -16 | -11 | -27 | -24 | 2 | -3 | 8 | 3 | -45 | 20 | -23 | 35 |
| ZANGOx ICMV IS 89305 | -5 | -5 | 0 | -2 | -5 | -9 | -5 | -5 | -6* | -6* | -3 | -2 | -2 | -4 | 7 | 5 | 73** | 63** | 40** | 31* | 2 | 1 | 9 | 4 | 33 | 20 | 95 | 71 | 6 | 12 | 8 | 25 | 6 | 6 | 10 | 7 | 4 | 38 | 18 | 41 |
| MORO x ICMV 167006 | -1 | -6 | -9 | -15 | -4 | -12 | -11 | -17 | -3 | 2 | -4 | -2 | 0 | -4 | 5 | -2 | 9 | 7 | 9 | 1 | 3 | 0 | 12 | 10 | 105** | 88* | 230** | 156* | -4 | 0 | 0 | 0 | 16 | 11 | 0 | -2 | 60 | 98 | -7 | 30 |
| Jirani x ICMV 167006 | 5 | -5 | 4 | -7 | 4 | -7 | 2 | -8 | -3 | 14** | -4 | 3 | 12 | -4 | 7 | -4 | 15 | 1 | 11 | -2 | -6 | -14 | 6 | -4 | 79 | 45 | 215* | 191 | -15 | 8 | -9 | 45 | 18* | 14 | 16 | 13 | -40 | 17 | -17 | -13 |
| HKP x ICMV 167006 | -11 | -21* | -7 | -15 | -9 | -15 | -20 | -26* | -2 | 1 | -5* | -5 | 11 | 11 | 6 | 5 | 17 | -8 | 1 | -20 | -2 | -4 | 11 | 6 | 121** | 94* | 291** | 237* | -9 | 15 | -6 | 45 | 15 | 4 | 13 | 7 | -46 | -22 | 64 | 86 |
| ZANGO x ICMV 167006 | -4 | -12 | 0 | -7 | -2 | -6 | 1 | -3 | -6* | -4 | -4 | -3 | 7 | 6 | 7 | 6 | 16 | -5 | 18 | 1 | 6 | 1 | 3 | -3 | 100* | 100* | 204* | 152 | 3 | 15 | -4 | 18 | 20* | 14 | 28** | 27* | 0 | 100 | -41 | -3 |
| Jirani x MORO | -1 | -6 | -8 | -12 | -5 | -9 | -10 | -14 | -8** | 1 | -8** | -4 | 3 | -8 | 4 | -1 | 13 | 1 | 7 | 3 | -1 | -13 | 4 | -4 | 60 | 22 | 235** | 176* | -6 | 25 | -14 | 36 | 2 | 2 | 5 | 4 | 56 | 314 | 37 | 82 |
| HKP x MORO | -1 | -17* | -8 | -21* | 0 | -14 | -7 | -18 | -4 | -2 | -4 | -2 | 12 | 7 | 10 | 4 | 2 | -21 | -7 | -31** | 9 | 3 | 2 | -2 | 46 | 39 | 139* | 111 | -25 | 0 | -18 | 27 | 6 | 0 | 8 | 5 | -11 | 65 | 52 | 84 |
| ZANGO x MORO | 2 | -11 | 0 | -13 | -6 | -16 | 3 | -7 | -8** | -5 | -2 | 1 | 6 | 0 | 11 | 4 | 14 | -7 | 14 | -9 | 5 | -3 | 2 | -3 | 39 | 28 | 22 | 12 | 0 | 17 | 4 | 27 | 6 | 6 | 15 | 12 | 209 | 371 | 43 | 63 |
| HKP x Jirani | 0 | -19** | -9 | -25** | -6 | -21* | -6 | -20* | -1 | 11** | -5* | 1 | 9 | -6 | 9 | -1 | 7 | -23 | 9 | -22* | 4 | -3 | 9 | 3 | 51 | 11 | 154 | 134 | -5 | -5 | -19 | -17 | 19* | 12 | 21* | 19 | -14 | 11 | 45 | 57 |
